# Factors influencing the outcome of integrated therapy approach in schizophrenia: A narrative review of the literature

**DOI:** 10.3389/fpsyt.2022.970210

**Published:** 2022-08-30

**Authors:** Giulia M. Giordano, Francesco Brando, Pasquale Pezzella, Maria De Angelis, Armida Mucci, Silvana Galderisi

**Affiliations:** University of Campania Luigi Vanvitelli, Naples, Italy

**Keywords:** antipsychotics, psychosocial interventions, side effects, treatment adherence, cognitive impairment, negative symptoms

## Abstract

The integration of pharmacotherapy with psychosocial interventions has an important role to play in the improvement of functional outcome of subjects with schizophrenia (SCZ), in all stages of the disorder. It is essential for the adequate management of unmet therapeutic needs, such as negative symptoms and cognitive dysfunctions which account for most of the functional impairment of subjects with SCZ and do not respond to available antipsychotics. Enhancing the knowledge on factors involved in the effectiveness of integrated treatment plans is an important step forward for SCZ care. This review aims to identify factors that might influence the impact of integrated treatments on functional outcome. Most studies on the impact of psychosocial treatments on functional outcome of subjects with SCZ did not control for the effect of prescribed antipsychotics or concomitant medications. However, several factors relevant to ongoing pharmacological treatment might influence the outcome of integrated therapy, with an impact on the adherence to treatment (e.g., therapeutic alliance and polypharmacotherapy) or on illness-related factors addressed by the psychosocial interventions (e.g., cognitive dysfunctions or motivational deficits). Indirect evidence suggests that treatment integration should consider the possible detrimental effects of different antipsychotics or concomitant medications on cognitive functions, as well as on secondary negative symptoms. Cognitive dysfunctions can interfere with participation to an integrated treatment plan and can be worsened by extrapyramidal or metabolic side effects of antipsychotics, or concomitant treatment with anticholinergics or benzodiazepines. Secondary negative symptoms, due to positive symptoms, sedation, extrapyramidal side effects or untreated depression, might cause early drop-out and poor adherence to treatment. Researchers and clinicians should examine all the above-mentioned factors and implement appropriate and personalized integrated treatments to improve the outcome of SCZ.

## Introduction

Schizophrenia (SCZ) is a complex and severe mental disorder. Evidence suggest that its clinical phenotype is the result of the interaction among genetic, biological, psychological, social and environmental factors. As a matter of fact, SCZ clinical phenotypes are very heterogeneous in terms of psychopathological features, risk factors, comorbidities, response to treatment and personal developmental trajectories (1–11).

The disorder is ranked among the 15 leading causes of disability worldwide (12): subjects with schizophrenia show a shorter lifespan than the average general population, are at increased risk of suicide, and of physical health problems (e.g., cardiovascular and lung illnesses, cancers, and obesity) and have a poor quality of life and functional outcome (2, 13–19), with only 15% of patients reaching criteria for clinical recovery (20–22).

The impairment in functional outcome includes deficits in self-care, interpersonal relationships, everyday life skills and work abilities, even during periods of remission from active psychosis (5, 13, 14, 16, 23–27). Several variables, some related to the illness, others to personal resources, and others to the context in which the person lives, seem to influence the functional outcome of subjects with schizophrenia, through direct or indirect relationships (5, 13, 14, 16, 26–28). Among the illness-related variables, negative symptoms, deficits in cognitive functions (neurocognition and social cognition) and functional capacity account for most of the functional impairment of patients and still represent unmet therapeutic needs (5, 16, 25–27, 29). These aspects are present since the onset and the early stages of the disorder, often predate it and show an elevated stability along the course of the illness (30–35).

The personal resources refer to resilience, coping strategies, healing styles and self-esteem; context-related variables include socio-economic status of the family, financial and employment opportunities, family and social incentives, stigma and social network (5, 13, 14, 16, 25–27, 36).

The relationships among these variables and functional outcome seem to be very complex and to follow multiple and complicated pathways (5, 16, 26, 27, 37). For instance, it has been found that neurocognition influences the functional outcome, in particular the areas of everyday and work skills, mainly indirectly through functional capacity, social cognition, engagement with services, and internalized stigma, which in turn is indirectly associated with the functional outcome through the resilience (16, 27). Negative symptoms belonging to the Experiential domain (avolition, anhedonia, and asociality), influence the functional outcome, in particular the area of interpersonal relationships, both directly and indirectly, through internalized stigma, resilience and engagement with services (16, 27).

Furthermore, it has been found, by using network analyses, that functional capacity and everyday life skills emerge as the most central and interconnected nodes (5, 26, 37). Functional capacity is the bridge between cognition (neurocognition and social cognition) and functional outcome (5, 26, 37), in particular the area of everyday life skills which, in its turn, connects other domains of functional outcome with psychopathology and internalized stigma (5, 26). The two domains of negative symptoms (the Experiential domain and the Expressive one) have different associations with functional outcome: the former one, defined above, with interpersonal relationships and work skills, while the latter domain, consisting of blunted affect and alogia, is connected with everyday life skills (5, 26, 37).

Understanding the complex relationships among multiple factors and functional outcome may foster the implementation of recovery-oriented and integrated treatment programs (psychosocial and pharmacological), that might be individualized according to patient's needs, to reduce symptom severity and improve patient's functional outcome, thus increasing the possibility of recovery (38–41). To date, the use of antipsychotics represents the main treatment strategy to provide patients with an effective symptom control and clinical stability (38–42). However, pharmacological treatments are most effective at reducing positive symptoms, but do not target adequately some aspects of schizophrenia, such as negative symptoms, cognitive impairment, and functional outcome. In addition, medication adherence is often poor (42–44), leading to high rates of relapse and hospitalization. Therefore, psychosocial interventions are usually provided in addition to pharmacotherapy and play an important role in the treatment of schizophrenia, in particular in improving functional outcome.

Different modalities and combinations of psychosocial interventions have been developed with the aim to address the complex individualized needs of patients (38, 40, 45), including the management of negative symptoms and cognitive dysfunctions (38–41, 46–48), to improve patient's reintegration in the community, promote recovery, and support patients and their families (38, 40, 41, 49, 50). Some psychosocial interventions, such as psychoeducation and cognitive behavioral therapy (CBT), allow patients to gain knowledge about their illness; others, such as cognitive remediation, social skills training, and supported employment may enhance adaptive skills (45). These interventions are briefly described below.

### Psychoeducation

Psychoeducation is an intervention that aims to provide patients and their families with information about the disease and its treatment, as well as disease management problem-solving and coping skills and to access community mental health-care services. It allows to establish a collaborative relationship between mental health providers, patients and their caregivers, helping them to reduce the burden of the disease and develop recovery strategies (40). This intervention is effective in reducing relapses, hospitalization, stigma and increasing patient's adherence to treatment and has an impact on various functional determinants, such as resilience, stigma, active participation in shared decision-making, ability to ask for service help when necessary, thus improving their quality of life and the overall functioning (38, 40, 45).

### Cognitive behavioral therapy

CBT aims to modify dysfunctional beliefs, by helping subjects to understand the link between perceptions, beliefs, emotional and behavioral reactions. This intervention has proven effective in reducing the intensity of or distress related to positive symptoms and improving overall functioning (6, 38, 40, 45), including work and social functioning (51–54).

### Cognitive remediation

Cognitive remediation for schizophrenia is a behavioral training-based intervention aimed to determine an improvement of individual's cognitive functions (51, 52). Several different interventions have been developed; they can have an individual or group format, use pen and paper or computerized modalities, including bottom-up or top-down strategies or include different combinations of these elements and can also vary in the frequency of sessions and the duration of treatment programs (55). Cognitive remediation usually utilizes exercises aimed at improving cognitive deficits in clinically stable patients with schizophrenia and has found to have a consistent small-to-moderate positive effect on cognitive functions (mainly attention, executive functions, social cognition, and metacognition) (38, 40, 45, 56–60). It has a positive effect also on work and social functioning (60), probably mediated by improvement in cognitive domains (58, 59, 61), particularly when it is combined with other rehabilitation techniques, such as psychoeducation and training to develop social, vocational and daily living skills (45). In addition, this intervention has demonstrated efficacy in improving negative symptoms (29, 60, 62–65).

### Social skills training

Social Skills Training is a psychosocial therapy that is based on the discussion of the contextual (setting), non-verbal (body language), and paralinguistic (voice tone) aspects of interpersonal interactions (66, 67). It is an intervention of proven effectiveness to improve social skills in subjects with schizophrenia (38, 40, 45). It has an impact also on cognitive deficits, negative symptoms and psychosocial functioning (29, 64, 66, 68, 69) and has proved effective when associated with cognitive remediation or supported employment (45).

### Supported employment interventions

Furthermore, taking into account that subjects with schizophrenia are 6–7 times more likely to be unemployed than those without such disorders, supported employment interventions, especially when integrated with cognitive remediation and social skills training, might be implemented to help patients to obtain competitive employment, work longer and have higher salaries than persons without such support (38, 40).

### Early intervention

A meta-analysis of 10 randomized clinical trials was conducted in the early phases of schizophrenia with the aim to compare integrated treatment (different modalities of psychosocial interventions in conjunction with antipsychotics) with “pharmacological treatment as usual”. This study has demonstrated that early integrated treatment was better than “treatment as usual” in favoring control of symptoms, reduction of relapses and hospitalization rates, adherence to treatment, involvement of the subject in school or work activities, and improvement of the quality of life and global functioning (70). Therefore, early integration of psychosocial interventions with pharmacological treatments should be encouraged (71).

### Factors influencing the outcome of integrated treatment in schizophrenia

However, despite the recognition of the advantages of early, individualized and integrated treatment plans to improve patient outcomes, some difficulties might be encountered in the implementation of evidence-based integrated treatment protocols in clinical settings. For instance, predictors of treatment response are still unavailable. Failure to achieve progress in this direction might be due to the lack of control for potential confounding factors: in fact, in most studies, antipsychotics or concomitant medications prescribed to patients participating in a clinical trial have been typically designated as “standard treatment” or “treatment as usual”, and not systematically evaluated (39, 72). However, several factors relevant to the ongoing pharmacological treatment might influence the outcome of integrated therapy, with an impact on the adherence to treatment (e.g., therapeutic alliance and polypharmacotherapy) or on illness-related factors addressed by the psychosocial interventions (e.g., cognitive dysfunctions or negative symptoms). The integration of treatments should consider the possible detrimental effects of different antipsychotics or concomitant medications on cognitive functions, as well as on secondary negative symptoms (38, 39, 42, 73). Cognitive dysfunctions can be worsened by extrapyramidal (74, 75) or metabolic side effects of antipsychotics (76), or concomitant treatment with anticholinergics or benzodiazepines (77–79). Extrapyramidal side effects, sedation, positive symptoms, or untreated depression might cause secondary negative symptoms and lead to poor adherence to treatment (38, 42, 80).

**Figure 1:**
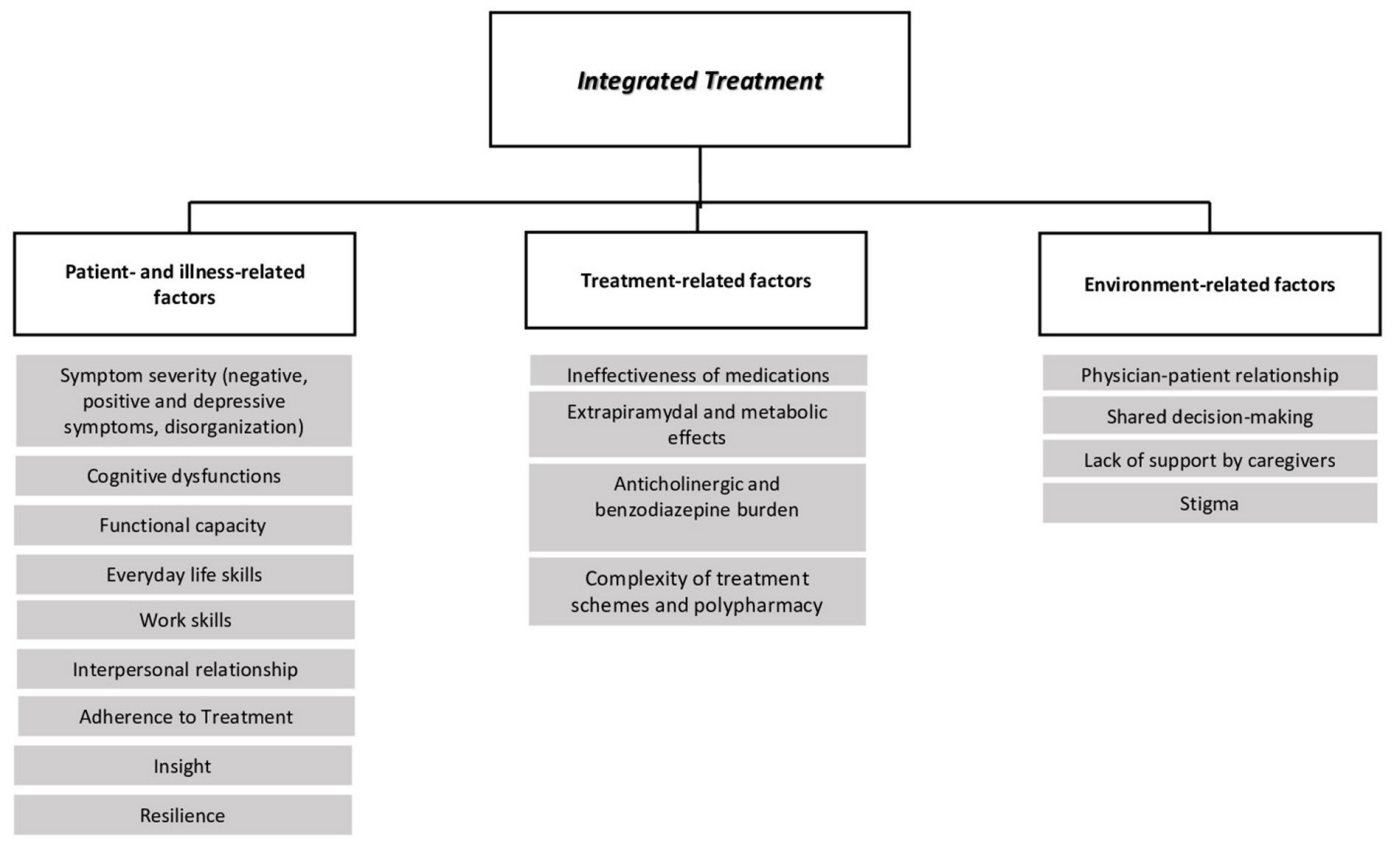
Factors influencing the impact of integrated treatment on functional outcome.

In this manuscript we will provide clinicians with an overview of factors that might influence the impact of integrated pharmacological and psychosocial treatments on functional outcome (Figure 1). We will highlight areas of interaction between treatments with potential confounding factors, including medication side effects, patient adherence to treatment, cognitive dysfunctions and negative symptoms.

## Methodology

For this review, we searched PubMed for articles in English published until January 2022, including the following terms: [(psychosis OR schizophrenia OR “Psychotic disorders”) AND (SGA OR “second generation antipsychotics” OR FGA OR “first generation antipsychotics” OR psychopharmacology OR antipsychotics) AND (“psychosocial rehabilitation” OR “psychosocial interventions”)] OR [(psychosis OR schizophrenia OR “Psychotic disorders”) AND “integrated treatment”]. We focused on five reviews and one meta-analysis. In addition, additional hand-searching was performed to identify publications relevant to the topic that were missed by the search strategy. Therefore, 32 reviews and 29 meta-analyses were included by hand search. Original landmark studies were selected from the reference list of reviews and meta-analyses.

## Pharmacotherapy

Antipsychotic treatment provides substantial benefits on symptom dimensions in schizophrenia spectrum disorders and represents the cornerstone of clinical stabilization, a condition in which psychotic symptoms are absent or of mild/moderate severity and require no treatment changes and/or hospital admissions. This condition is necessary to implement an effective integrated treatment plan (81). In fact, stable antipsychotic treatment is required to avoid symptom exacerbations and relapses (82), and implement non-pharmacological interventions, thus improving patients' outcomes (83–85). Antipsychotic treatment relies mostly on first generation (FGA) and second generation (SGA) antipsychotics. FGA are potent antagonists of dopamine D2 receptors (responsible for the antipsychotic effect), as well as adrenergic, cholinergic and histaminergic receptors (responsible for the high rates of side effects) (86). On the other hand, SGAs have a lower affinity for the aforementioned receptors, and they block the 5HT2A receptor (13), thus having reduced extrapyramidal side effects (86), but with comparable rates of adverse events such as sedation and weight gain, with respect to FGA (13).

Along with antipsychotic drugs, many individuals with schizophrenia require other types of medications, such as anticholinergics, benzodiazepines, antidepressants and mood stabilizers.

In most clinical trials evaluating the effectiveness of an integrated therapy, type and dosage of prescribed antipsychotics or concomitant medications have typically been designated as “standard treatment” or “treatment as usual” and not systematically evaluated (39, 72). However, some factors relevant to ongoing pharmacological treatment, such as the appearance of side effects, might impact different aspects, for instance adherence to treatment, cognitive functions, negative symptoms, and influence outcome. Within an integrated treatment plan, clinicians should carefully take into account the type and dosage of ongoing pharmacological treatments, in order to rule out the possible detrimental effects of pharmacotherapy on the outcome of the interventions.

### Pharmacological treatment side effects

Antipsychotics are associated with several side effects, sometimes severe ones, which can reduce adherence to treatment, thus leading to a reduction or loss of clinical stability, which in turn may affect the outcome of an integrated therapy (38, 87–90). The influence of side effects on treatment adherence, cognitive abilities and negative symptoms will be described more in depth in the paragraphs 4–6.

Common side effects are represented by motor symptoms and cardiometabolic effects. In general, first-generation antipsychotics (FGAs) are associated with motor side effects, while second-generation antipsychotics (SGAs) with weight gain and metabolic issues (75, 91–95).

Motor symptoms, commonly referred to as extrapyramidal symptoms (EPS), affect movement and muscle tone and can lead to parkinsonism (tremor, muscle stiffness and bradykinesia), muscle spasms (dystonia), subjective and objective restlessness (akathisia), involuntary and repetitive movements of the trunk, limbs, tongue and jaw, i.e., dyskinesia (96, 97). Literature widely reports a higher prevalence of EPS in subjects treated with FGAs, as compared to subjects treated with SGAs (75, 98–101). The higher incidence of EPS during treatment with FGAs, as compared to SGAs, could lead to poor treatment adherence and therefore compromise the clinical stability of the subject (75). In addition, EPS might worsen cognitive deficits and cause secondary negative symptoms in schizophrenia, thus indirectly influencing the outcome of an integrated treatment (29, 75, 102, 103).

The cardiometabolic effects include weight gain and metabolic syndrome, which refers to the presence of at least three out of the five following criteria: increased waist circumference, hypertriglyceridemia, reduced high-density lipoprotein (HDL), elevated blood pressure, and elevated fasting glucose (76, 92). It has been shown that metabolic effects can adversely affect cognitive performance in subjects without psychiatric disorders and also in subjects with schizophrenia (104–111), thereby worsening the outcome of an integrated treatment.

Other side effects to be ascribed to a different extent to all antipsychotics are represented by changes in cardiac electrical activity, hyperprolactinemia, anticholinergic adverse effects, sedation, decrease of the seizure threshold, sialorrhea, neutropenia and agranulocytosis, and neuroleptic malignant syndrome (112–115).

Besides being poorly tolerated by the patient, the presence of side effects seriously affects patient's general health, is associated with less favorable attitudes toward antipsychotic treatments and, consequently, leads to a reduction in adherence to treatment, thus compromising the efficacy of an integrated treatment (38, 76, 89, 116).

Besides the impact of antipsychotic drugs, other treatments can influence the outcome of an integrated treatment in subjects with schizophrenia. In particular, long-term co-treatment with benzodiazepines has been seen to impair cognitive abilities, both through a direct or indirect mechanism (mediated by the effects of their potential sedation) (76, 117–120).

Furthermore, long-term co-treatment with anticholinergics drugs, often used to reduce extrapyramidal symptoms, is associated with poor adherence to drug treatment (121) and with a worsening of pre-existing cognitive dysfunctions (78, 79, 117, 122–128), while the progressive dosage reduction of anticholinergics is accompanied by a positive effect on cognition (129).

In the light of the available evidence, when evaluating the outcome of a psychosocial intervention targeting for instance cognitive dysfunctions, it is important for clinicians to rule out the potential confounding effects of anticholinergic or benzodiazepine drug treatments on cognition. The influence of concomitant medication side effects on cognitive abilities will be described more in depth in paragraph 5.

## Adherence to treatment

Available evidence suggests that the access of patient to additional treatments other than pharmacotherapy is influenced by the clinical stability, defined as the absence of relapses, which require the hospitalization or substantial changes of ongoing pharmacological treatments (42, 130). By preventing relapses, the use of antipsychotics represents the main strategy to ensure clinical stability (38, 47). However, a poor treatment adherence still represents a major issue in the care of subjects with schizophrenia (38, 47, 59, 60), affecting about 50% of the patients (42–44). The lack of treatment adherence increases relapse and hospitalization rates (131); however, it is has been noted that it is difficult to state whether non-adherence is the cause of relapses and hospitalizations or whether it represents an indicator of disease progression (38, 132, 133).

The adherence to antipsychotics is influenced by several factors, some related to the patient (e.g., poor insight, neurocognitive dysfunctions, symptoms severity or lack of symptom improvement), some to medication (e.g., ineffectiveness of or adverse effects of medications, complexity of treatment schemes, and polypharmacotherapy) and some to patient's environment (e.g., lack of support by caregivers, poor physician-patient relationship, non-shared decision making, and lack of information about the disease and its treatment) (42, 73, 134).

Poor insight is regarded as one of the main causes of treatment non-adherence in subject with schizophrenia (42, 43, 73, 135–138). Indeed, individuals with a better awareness of their pathology have better attitudes toward drugs, thus leading to better adherence and therapeutic results (135). Conversely, individuals who are unaware of their condition will be less motivated to resolve their symptoms and will not need their symptoms to be managed, thus leading to poor therapeutic results and great likelihood of relapses (73, 135, 139). Poor insight and non-adherence to treatment appear to contribute directly to a worse response to integrated therapy, leading to a poor outcome (140).

Neurocognitive impairment can adversely affect adherence to treatment, as it impairs processes that are fundamental for adherence, such as attention and memory, leading to a difficulty in understanding or managing the programs of therapy, especially when the treatment schemes are very complex (141–144). However, some studies did not find a relationship between cognitive impairment and non-adherence to treatment (139, 145–152).

Lack of symptom improvement (due to the ineffectiveness of medications), with a worsening of positive and/or negative symptoms, might represent a potential factor for non-adherence to treatment (42, 80, 116, 153–155). However, some studies did not find an association between symptom severity and adherence rates (156, 157).

Furthermore, it has been reported that adverse effects of antipsychotics have an important role in the adherence to treatment. Indeed, they can lead to the discontinuation of therapy (38, 158, 159), due to patients' poor tolerability toward these effects (160).

In the general population, and particularly in individuals over the age of 65, polypharmacy is more frequently associated with non-adherence to treatment simply because of the large number of medications that subjects can forget to take daily (160–162). Also for subjects with schizophrenia, polypharmacy and the complexity of treatment schemes have been associated with non-adherence to therapy (38, 42, 43, 80, 163–170); in a recent study it was shown that the risk of non-adherence is about double in subjects who take more than one drug compared to those who take monotherapy (80, 171). However, it is also possible that polypharmacy indirectly affects adherence to treatment, due to an increase in the number of side effects (172, 173).

Adherence to treatment is favored by a good physician-patient relationship, through a better understanding of the individual motivation to treatment and by involving the patient in treatment plans and decisions (shared decision making). Indeed, the shared decision-making approach, taking into account patient's preferences, increases the motivation to treatment and, consequently, treatment adherence (174–183). In addition, it has been demonstrated that strong therapeutic relationships with a good therapeutic alliance are related to a decrease of symptom severity, hospitalizations, drop-out rates and to an increase in treatment adherence (48, 183, 184), while poor therapeutic relationships have been shown to increase the risk for non-adherence (40, 43, 185).

Within the integrated treatment approach, it is crucial to identify the possible causes that underlie non-adherence to treatment, exploring patient's individual needs, preferences, satisfaction, in order to establish an adequate treatment plan (40, 42, 186).

To improve adherence to treatment of subjects with schizophrenia spectrum disorders, it is preferable to (i) improve insight of patients with poor or no awareness of their disease, (ii) reduce negative attitudes toward drugs, (iii) improve cognitive functions, (iv) reduce symptom severity, (v) avoid, when possible, the use of polypharmacotherapy or complexity of treatment schemes, and (vi) involve patients in the treatment decision-making process through a good therapeutic alliance.

## Cognitive dysfunctions

Cognitive dysfunctions, which are defined as deficits of different cognitive domains (speed of processing, attention and vigilance, working memory, verbal learning and memory, visuospatial learning and memory, reasoning and problem solving, and social cognition) (187–191), represent a crucial domain to be targeted within an integrated treatment approach. They are present since the onset and the early stages of the disorder, often predate it and are associated to poor premorbid functioning, and show an elevated stability along the course of the illness (30, 33). Cognitive dysfunctions account for most of the functional impairment of patients, are poorly responsive to antipsychotic treatments, thus still represent an unmet therapeutic need (5, 16, 26, 27, 192–194). Different reviews and metanalyses reported that cognitive remediation therapy, skills-based training and physical exercise, represent good treatment strategies that might be used as part of an integrated treatment to improve cognitive impairment of subjects with schizophrenia (56–60, 69, 195–199). Other psychosocial interventions, such as cognitive behavioral therapy and psychoeducation, which have been shown to improve psychosocial functioning, caregiver's burden and measures related to overall wellbeing (psychoeducation), could be effective in improving cognitive dysfunctions, but more research is needed to properly assess their effectiveness on this domain of functioning (51–54, 192, 200–203).

Despite the reports of psychosocial interventions efficacy in ameliorating cognitive deficits, the treatment response rate is very heterogeneous (204). Some potential predictors of treatment response, such as participants' age, premorbid IQ, duration of illness and of treatment have been investigated in previous studies (204). The duration of the psychosocial intervention might also have an effect on outcome, and the number of training sessions and the overall duration of the interventions show a great degree of variation between studies, depending on the type of intervention (47). For cognitive remediation, it is recommended that the intervention should be of sufficient intensity and duration to determine an enduring improvement of cognitive functions (205). Most psychosocial interventions typically extend up to 6 months with 1-h sessions, 2–5 times a week (47). However, no systematic investigation of the duration has been carried out and more studies are needed in order to find out whether longer training sessions and a greater number of sessions lead to a further improvement of cognitive functions and better functional outcome and whether the influence of intensity and duration of training sessions varies according to the type of intervention (204). In addition, further investigation of predictors of response to treatment and potential confounding factors influencing the outcome is needed (204).

According to available meta-analyses SGAs were consistently superior to FGAs in improving cognitive functions, with no molecule consistently outperforming the others (194, 206–209). A recent meta-analysis (210) reported favorable effects for amisulpride, quetiapine, lurasidone, olanzapine, perphenazine, risperidone, sertindole, and ziprasidone, while remoxipride, clozapine and haloperidol, were outperformed by placebo in most cognitive domains (210). The positive impact of perphenazine on cognition was discussed as an unexpected result by the authors of the meta-analysis, and in need of further replications (211). Overall, SGAs have been shown to be more efficacious in improving cognitive functions, as compared to FGAs; however it is questionable whether this advantage reflects a real improvement of cognitive functions or a lower burden (as compared to FGAs) of extrapyramidal side effects that might worsen cognitive functions (212).

Besides the type of antipsychotic, also the dose seems to influence cognition in subjects with schizophrenia (76): higher antipsychotic doses and polypharmacy are associated with worse cognitive functioning, and an improvement in different cognitive domains (memory, visuospatial, language, attention, and delayed memory) has been observed when the antipsychotic dose was reduced (76). Higher antipsychotic dosage has been found in association with lower cognitive functions after a cognitive remediation program, suggesting that patients with a more severe illness (who generally receive high doses of antipsychotics) had less benefit from cognitive remediation, or that, high antipsychotic doses limit the effectiveness of the intervention, due to the presence of EPS (213, 214). In fact, clinical practice and data collected from the literature show that EPS might worsen neurocognitive (74, 75, 215–221) and social cognition functions (75), thus influencing the outcome of a psychosocial intervention targeting them (74, 75, 215–221). Indeed, subjects with EPS had worse overall cognitive performance, with lower scores for attention, motor skills and verbal learning domains (74, 215–217, 219). Studies that focused only on tardive dyskinesia reported inconsistent results, since two studies found a relationship between tardive dyskinesia and deficits in spatial working memory, visuospatial skills, and attention (220, 221), while another study did not find any significant association between tardive dyskinesia and cognitive impairment (222).

To date, it is not clear which is the mechanism that underlies the relationship between EPS and neurocognitive deficits; however, some theories have been developed: subjects that develop EPS, as compared to subjects that do not develop EPS, may have pre-existing neural dysfunctions, which could explain both greater vulnerability to develop EPS and worse cognitive performance after treatment with antipsychotics (223), or EPS might affect those motor skills required to perform specific neurocognitive tasks. Indeed, Fervaha et al. demonstrated that subjects with schizophrenia and EPS had a lower cognitive composite score than subjects without EPS, but this result was not confirmed after controlling for the effect of motor speed (74). However, this hypothesis explains the association between EPS and performance of only those cognitive tasks involving timed tests or motor movements, but not the others (e.g., verbal learning and social cognition) (75).

The metabolic syndrome is present in about 33.5% of subjects with schizophrenia. Different risk factors are associated with the appearance of this syndrome: lifestyle factors, such as smoking, poor eating habits and sedentary behavior, antipsychotic medications, as well as genetic factors relevant to schizophrenia and/or to treatment response that overlap with genes related to metabolic functions (224). Most studies reported an association between metabolic effects and cognitive dysfunctions, both in subjects without psychiatric disorders (104–108) and in those with schizophrenia (76, 109–111). In particular, in subjects with schizophrenia, the metabolic syndrome seems to influence negatively some cognitive functions, such as processing speed, memory, attention, and reasoning and problem solving (109–111). Conversely, other studies failed to find this association (225–227). However, some of these studies, with the exception of the CATIE trial, have found that specific aspects of metabolic syndrome, such as hypertension, were correlated with lower cognitive scores, whereas other factors, such as increased waist circumference and dyslipidemia were less consistently associated with general impairments of cognition in schizophrenia (226, 227). Furthermore, in one study, hyperglycemia was associated with better verbal memory performance (226). Further studies examining the associations between individual metabolic symptoms and cognition in subjects with schizophrenia are needed to draw sound conclusions (76).

The anticholinergic burden has been related to the risk of cognitive impairment both in elderly subjects and in subjects with schizophrenia (78, 79, 228–230). Several large and well-conducted studies have shown an association between the use of drugs with anticholinergic properties (some antipsychotics and anticholinergic drugs) and poor cognitive performance, with the anticholinergic burden increasing cognitive impairment in specific domains (e.g., processing speed, attention, language, problem solving, and psychomotor performance) (78, 123–125, 127, 231–235). Another study showed that exposure to drugs with anticholinergic properties impairs attention and declarative memory in schizophrenia, but has no effect on other aspects of cognition, including intelligence, working memory, executive functioning, and motor speed (236). One study detected the possible impact of the anticholinergic burden on the outcome of a cognitive remediation program, and found that the serum level of anticholinergic activity, measured by radioreceptor assay, at baseline negatively predicted cognitive remediation-induced changes, indicating that this variable had a negative impact on the patient's response to the intervention (235).

Patients with schizophrenia are often prescribed benzodiazepines as adjuvant drugs. Long-term use of these drugs has been shown to negatively impact cognition, and adverse effects have been reported on specific cognitive abilities, such as attention and working memory (77, 120). Benzodiazepines represent a specific factor contributing to worsening of cognitive functions not only in individuals with schizophrenia, but also in the elderly (237) and progressive reduction in benzodiazepine dosage has been associated with improved cognitive functions (118, 119).

As part of an integrated treatment aimed at improving cognitive functions, clinicians should take into account some factors that could influence cognitive abilities, such as the type and dosage of used drugs, adherence to treatment and pharmacotherapy side effects. To avoid a negative impact on cognitive functions, clinicians should favor the use of molecules that have a low impact on these functions and limit the burden of anticholinergics and benzodiazepines, particularly in long-term treatment (77–79).

## Negative symptoms

Negative symptoms are a complex and heterogeneous construct with a pivotal role in the care of subjects with schizophrenia. They are present since the onset and the early stages of the disorder, often predate it, are associated to poor premorbid functioning and functional outcome, and show an elevated stability along the course of the illness (31, 32, 238).

Negative symptoms include avolition, anhedonia and asociality, which cluster into the Experiential domain, and blunted affect and alogia, which cluster into the Expressive Deficit domain (102, 103, 203, 239–241). Different pathophysiological features and different associations with patient's functioning have been found for the two negative symptom domains, thus implicating potential different treatment strategies for them (5, 16, 26, 27, 102, 103, 242–247). To date, there are no pharmacological treatments of proven efficacy for negative symptoms, particularly for primary and persistent ones, which still remain an unmet need in the care of subjects with schizophrenia (29, 103, 248–251).

Different reviews and metanalyses reported that some psychosocial interventions, such as cognitive training and skills-based training, represent a good treatment strategy that might be used as part of an integrated treatment to improve negative symptoms (29, 60, 62–66, 68). The potential role of cognitive behavioral therapy in improving negative symptoms is not yet clarified, since most meta-analyses have found small or insignificant effects of CBT on negative symptoms (29). Overall, different sources of heterogeneity in studies focusing on psychosocial interventions should be considered. Indeed, most studies included in the above-cited metanalyses did not have negative symptoms as a primary outcome, and few studies defined a minimum threshold for negative symptoms at inclusion. Patient populations were small and very heterogeneous in terms of illness and treatment duration, as well as of psychopathological features. In addition, most studies in the meta-analyses compared the psychosocial intervention to “treatment as usual”, without providing information on type and dosage of antipsychotics. Finally, most studies used a global/total score for the evaluation of negative symptoms and did not report specific information on the characteristics of these symptoms, most importantly they did not distinguish between primary and secondary negative symptoms.

Indirect evidence suggests that different confounding factors might influence the outcome of integrated treatments targeting negative symptoms, in particular the type of antipsychotic, as well as factors that might cause secondary negative symptoms (parkinsonism, positive symptoms, or depression). Therefore, before starting an integrated treatment plan, also aimed at improving negative symptoms, clinicians should be encouraged to carefully rule out the effects of these confounding factors.

As to antipsychotic drugs, it has been demonstrated that different types of antipsychotics have a different impact on negative symptoms in subjects with schizophrenia (13, 29, 252). Amisulpride and cariprazine showed promising results in targeting predominant negative symptoms (253–255). Furthermore, a meta-analysis conducted by Fusar-Poli et al. (252) found a small improvement in negative symptoms with SGAs, but not with FGAs. Within the SGA, amisulpiride, clozapine, olanzapine, quetiapine, and risperidone have been shown to be more effective than FGAs in ameliorating negative symptoms (13). However, in these metanalyses, included clinical data were very heterogenous, and no specific information on the characteristics of negative symptoms, for instance on the differentiation between primary and secondary negative ones, was provided (29). Distinguishing between primary and secondary negative symptoms is an important clinical issue from a therapeutic and a prognostic point of view. Indeed, while primary negative symptoms tend to be persistent and treatment resistant (256, 257), secondary negative symptoms might be effectively treated, targeting the underlying factors (e.g., parkinsonism, positive symptoms, or depression) (102, 103). These factors represent possible confounders when evaluating the outcome of an integrated treatment plan targeting negative symptoms.

Antipsychotic drugs, for instance, and in particular FGAs as reported in the paragraph 3, may induce extrapyramidal side effects. Some of these side effects, in particular bradykinesia and hypomimia, might mimic expressive negative symptoms. Furthermore, excessive sedation can lead to decreased activity and/or amotivation. In these cases, the differential diagnosis is made through a longitudinal observation of the symptoms. An increase in the severity of negative symptoms following an increase in the dose of the drug suggests the presence of secondary negative symptoms. In addition, a standard clinical examination to evaluate the presence of other EPS, such as tremor or rigidity, may be useful to rule out or diagnose drug-induced parkinsonism. The presence of negative symptoms secondary to extrapyramidal effects or sedation due to antipsychotic treatment should be treated with a possible dose reduction or drug change, choosing antipsychotics with lower risk of EPS and sedation (29).

Negative symptoms may result from positive ones; for instance, patients' experiences of threat and fear due to persecutory thoughts or hallucinations can lead to social withdrawal. To assess the secondary nature of negative symptoms, clinicians should observe whether negative symptoms occur during periods of psychotic exacerbation or as a result of changes in pharmacotherapy, or whether these symptoms improve as hallucinations and delusions are ameliorated by the antipsychotic treatment. In the presence of negative symptoms secondary to positive symptoms it is appropriate to re-evaluate the antipsychotic treatment with respect to the dosage or type of antipsychotic (29, 258–260).

Due to the overlap between depression and negative symptoms, it is often difficult to distinguish between primary negative symptoms, secondary negative symptoms due to depression, and depression without negative symptoms (261, 262). Furthermore, the inability to express internal experiences and emotions in patients with schizophrenia can hinder the detection of depression (261). To assess the secondary nature of negative symptoms, clinicians should observe whether negative symptoms, e.g., the social withdrawal, might be induced by depression. In this case, the presence of sadness, hopelessness and/or guilt might suggest that the negative symptomatology is secondary to depression (263, 264). After detecting depressive symptoms, the use of an antipsychotic drug with antidepressant properties (e.g., quetiapine, amisulpride, cariprazine, aripiprazole, clozapine, and olanzapine), or the addition of an antidepressant drug and/or of cognitive-behavioral therapy should be considered (13, 29, 265–267).

In the clinical practice, the distinction between primary and secondary negative symptoms can be challenging and should be considered as part of the assessment. It may require a longitudinal observation which is not always feasible. Indeed, to evaluate the secondary nature of negative symptoms, clinicians should observe whether negative symptoms occur during periods of psychotic exacerbation or in concomitance with depressive symptoms or following changes in pharmacotherapy.

Therefore, as part of an integrated treatment aimed at improving negative symptoms, clinicians should rule out the effects of some confounding factors, such as the dose and the type of drug used, parkinsonism, positive symptoms, or depression (29, 102, 103, 261, 268–270). To allow a good management of negative symptoms, clinicians should optimize the psychopharmacologic treatment to control positive and depressive symptoms, as well as to limit the appearance of EPS, thus favoring the outcome of the integrated treatment (29).

## Discussion

Within the recovery-oriented approach in the care of subjects with schizophrenia, the need of integrated therapy, involving pharmacological treatment and psychosocial interventions, is widely recognized (38–41). Some important implications for the implementation of evidence-based practice for guiding integrated therapy in clinical settings emerged from the review of the literature reported in this paper.

First, antipsychotics represent the main treatment strategy to provide patients with an effective symptom control and clinical stability (38–42), which appears to represent a crucial condition to realize a structured and effective integrated treatment plan (81). In fact, stable antipsychotic treatment is required to avoid symptom exacerbations and relapses (82); clinical stability facilitates patient's engagement in non-pharmacological interventions, and increases the likelihood of a good outcome (83–85).

Second, psychosocial interventions are important to address the complex individual needs of patients, (38–41, 46–48), ameliorate negative symptoms and cognitive deficits, improve patient reintegration into the community, favor the patient's employment and support patients and their families, thus promoting recovery (38, 40, 41, 49, 50). In addition, through a good therapeutic alliance, psychosocial interventions improve the patient's insight in the illness, favor the adherence to treatment plans, thus reducing the risk of drug discontinuation and relapses.

Third, effective psychosocial interventions, such as cognitive training and skills-based training, should be made available to subjects with cognitive impairment, poor social competence, and negative symptoms. Such interventions should be provided as early as possible given the fact that the above-reported impairments have been described since early stages of the illness and even before clinical onset (30–35). In addition, psychoeducation and CBT should be implemented in clinical practice since they improve treatment adherence and reduce relapses and the distress related to positive symptoms. The impact of these interventions on functional outcome seems to be improved by combining different treatment approaches, basing on patient's individual needs and issues and the stage of the disease: cognitive remediation combined with psychoeducation and training to develop social, vocational and daily living skills; or social skills training combined with cognitive remediation and supported employment (45).

Fourth, besides their positive effects on symptoms control, antipsychotics and concomitant medications may induce adverse effects, that might seriously affect patient's general health, favor negative attitudes toward treatment and, consequently, lead to a reduction in the treatment adherence, thus compromising the efficacy of an integrated treatment (38, 76–79, 89, 116). Given the presence of differences across the available drugs in terms of individual response and tolerability, the characterization of the individual patient with respect to psychopathological dimensions, physical health and comorbidities should guide the treatment choice (6, 271, 272).

Fifth, patient-focused perspectives in the treatment plans, individual preferences, and satisfaction, should be taken into account in order to promote treatment adherence, improve long-term outcomes and facilitate the implementation of personalized treatment for subjects with schizophrenia, by evaluating individual needs (175, 178).

Sixth, understanding the complex relationships among multiple factors and functional outcome may foster the development of new treatments and the personalization of integrated psychosocial and pharmacological treatment programs. Therefore, the assessment of different variables, some related to the illness, others to personal resources and others to the context, should be encouraged and implemented within the clinical practice, in order to understand the individual patient needs and to establish a recovery-oriented treatment plan.

Seventh, every effort should be made to conduct clinical trials with standardized and replicable methodology. In addition, it will be important that such studies are specifically designed to clarify the impact of multiple confounding factors on patient's outcome, as well as to clarify the points of interactions between different types of treatment (pharmacological and psychosocial treatments) on different schizophrenia domains, and in particular on those which still represent unmet therapeutic needs.

However, the above considerations should be read in the light of the limitations of the studies included in this review. Most information gathered with respect to the interactions between pharmacological and psychosocial treatment was inferred indirectly from articles in which this topic was not the main focus of the study.

In conclusion, further studies are needed to clarify the impact of different variables on the outcome of an integrated treatment plan. A better understanding of possible predictors of treatment response, or of factors that might influence the patient's outcome, could enhance the implementation of personalized and individualized recovery-oriented treatments in clinical settings (6, 273, 274).

## Author contributions

GG, FB, MD, and PP performed the literature search. GG, FB, and PP drafted the article. AM and SG critically revised the work. All authors contributed to the article and approved the submitted version.

## Conflict of interest

Author SG has been a consultant and/or advisor to or has received honoraria from Millennium Pharmaceutical, Innova Pharma–Recordati Group, Janssen Pharmaceutica NV, Gedeon Richter-Recordati, Angelini, Lundbeck Italia, and Sunovion Pharmarmaceuticals. Author AM has been a consultant and/or advisor to or has received honoraria from Gedeon Richter Bulgaria, Janssen Pharmaceuticals, Lundbeck, Otsuka, Pfizer, and Pierre Fabre. The remaining authors declare that the research was conducted in the absence of any commercial or financial relationships that could be construed as a potential conflict of interest.

## Publisher's note

All claims expressed in this article are solely those of the authors and do not necessarily represent those of their affiliated organizations, or those of the publisher, the editors and the reviewers. Any product that may be evaluated in this article, or claim that may be made by its manufacturer, is not guaranteed or endorsed by the publisher.

## References

[1] Owen MJ Sawa A Mortensen PB. Schizophrenia. Lancet. (2016) 388:86–97. 10.1016/S0140-6736(15)01121-6 26777917 PMC4940219

[2] Howes OD McCutcheon R Agid O de Bartolomeis A van Beveren NJ Birnbaum ML. Treatment-resistant schizophrenia: treatment response and resistance in psychosis (TRRIP) working group consensus guidelines on diagnosis and terminology. Am J Psychiatry. (2017) 174:216–29. 10.1176/appi.ajp.2016.16050503 27919182 PMC6231547

[3] Stilo SA Murray RM. Non-genetic factors in schizophrenia. Curr Psychiatry Rep. (2019) 21:100. 10.1007/s11920-019-1091-3 31522306 PMC6745031

[4] Correll CU Schooler NR. Negative symptoms in schizophrenia: a review and clinical guide for recognition, assessment, and treatment. Neuropsychiatr Dis Treat. (2020) 16:519–34. 10.2147/NDT.S225643 32110026 PMC7041437

[5] Galderisi S Rucci P Mucci A Rossi A Rocca P Bertolino A. The interplay among psychopathology, personal resources, context-related factors and real-life functioning in schizophrenia: stability in relationships after 4 years and differences in network structure between recovered and non-recovered patients. World Psychiatry. (2020) 19:81–91. 10.1002/wps.20700 31922687 PMC6953544

[6] Maj M van Os J De Hert M Gaebel W Galderisi S Green MF. The clinical characterization of the patient with primary psychosis aimed at personalization of management. World Psychiatry. (2021) 20:4–33. 10.1002/wps.20809 33432763 PMC7801854

[7] Santo-Angles A Salvador R Gomar JJ Guerrero-Pedraza A Ramiro N Tristany J. Interindividual variability of functional connectome in schizophrenia. Schizophr Res. (2021) 235:65–73. 10.1016/j.schres.2021.07.010 34329851

[8] Gaebel W Zielasek J. Schizophrenia in 2020: trends in diagnosis and therapy. Psychiatry Clin Neurosci. (2015) 69:661–73. 10.1111/pcn.12322 26011091

[9] Gaebel W Riesbeck M Larach VW Falkai P Zielasek J. Trends in schizophrenia diagnosis and treatment. In: Javed A, Fountoulakis KN, editors. Advances in Psychiatry. Cham: Springer International Publishing (2019). p. 603–19.

[10] McCutcheon RA Krystal JH Howes OD. Dopamine and glutamate in schizophrenia: biology, symptoms and treatment. World Psychiatry. (2020) 19:15–33. 10.1002/wps.20693 31922684 PMC6953551

[11] Perrottelli A Giordano GM Brando F Giuliani L Mucci A. EEG-based measures in at-risk mental state and early stages of schizophrenia: a systematic review. Front Psychiatry. (2021) 12:653642. 10.3389/fpsyt.2021.653642 34017273 PMC8129021

[12] GBD 2016 Disease and Injury Incidence and Prevalence Collaborators. Global, regional, and national incidence, prevalence, and years lived with disability for 328 diseases and injuries for 195 countries, 1990–2016: a systematic analysis for the Global Burden of Disease Study 2016. Lancet. (2017) 390:1211–59. 10.1016/S0140-6736(17)32154-2 28919117 PMC5605509

[13] Leucht S Corves C Arbter D Engel RR Li C Davis JM. Second-generation versus first-generation antipsychotic drugs for schizophrenia: a meta-analysis. Lancet. (2009) 373:31–41. 10.1016/S0140-6736(08)61764-X 19058842

[14] Harvey PD Strassnig M. Predicting the severity of everyday functional disability in people with schizophrenia: cognitive deficits, functional capacity, symptoms, and health status. World Psychiatry. (2012) 11:73–9. 10.1016/j.wpsyc.2012.05.004 22654932 PMC3363376

[15] Fleischhacker WW Arango C Arteel P Barnes TR Carpenter W Duckworth K. Schizophrenia–time to commit to policy change. Schizophr Bull. (2014) 40(Suppl. 3):S165–94. 10.1093/schbul/sbu006 24778411 PMC4002061

[16] Galderisi S Rossi A Rocca P Bertolino A Mucci A Bucci P. The influence of illness-related variables, personal resources and context-related factors on real-life functioning of people with schizophrenia. World Psychiatry. (2014) 13:275–87. 10.1002/wps.20167 25273301 PMC4219069

[17] Green MF Horan WP Lee J McCleery A Reddy LF Wynn JK. Social disconnection in schizophrenia and the general community. Schizophr Bull. (2018) 44:242–9. 10.1093/schbul/sbx082 28637195 PMC5814840

[18] Harvey PD Strassnig MT Silberstein J. Prediction of disability in schizophrenia: symptoms, cognition, and self-assessment. J Exp Psychopathol. (2019) 10:2043808719865693. 10.1177/204380871986569330683165

[19] Saha S Chant D McGrath J. A systematic review of mortality in schizophrenia: is the differential mortality gap worsening over time? Arch Gen Psychiatry. (2007) 64:1123–31. 10.1001/archpsyc.64.10.1123 17909124

[20] Jääskeläinen E Juola P Hirvonen N McGrath JJ Saha S Isohanni M. A systematic review and meta-analysis of recovery in schizophrenia. Schizophr Bull. (2013) 39:1296–306. 10.1093/schbul/sbs130 23172003 PMC3796077

[21] Zipursky RB Agid O. Recovery, not progressive deterioration, should be the expectation in schizophrenia. World Psychiatry. (2015) 14:94–6. 10.1002/wps.20194 25655164 PMC4329903

[22] Vita A Barlati S. Recovery from schizophrenia: is it possible? Curr Opin Psychiatry. (2018) 31:246–55. 10.1097/YCO.0000000000000407 29474266

[23] Leung WW Bowie CR Harvey PD. Functional implications of neuropsychological normality and symptom remission in older outpatients diagnosed with schizophrenia: a cross-sectional study. J Int Neuropsychol Soc. (2008) 14:479–88. 10.1017/S1355617708080600 18419846 PMC2562528

[24] Harvey PD. Assessment of everyday functioning in schizophrenia: implications for treatments aimed at negative symptoms. Schizophr Res. (2013) 150:353–5. 10.1016/j.schres.2013.04.022 23668973 PMC3825780

[25] Galderisi S Rossi A Rocca P Bertolino A Mucci A Bucci P. Pathways to functional outcome in subjects with schizophrenia living in the community and their unaffected first-degree relatives. Schizophr Res. (2016) 175:154–60. 10.1016/j.schres.2016.04.043 27209527

[26] Galderisi S Rucci P Kirkpatrick B Mucci A Gibertoni D Rocca P. Interplay among psychopathologic variables, personal resources, context-related factors, and real-life functioning in individuals with schizophrenia: a network analysis. JAMA Psychiatry. (2018) 75:396–404. 10.1001/jamapsychiatry.2017.4607 29450447 PMC5875306

[27] Mucci A Galderisi S Gibertoni D Rossi A Rocca P Bertolino A. Factors associated with real-life functioning in persons with schizophrenia in a 4-year follow-up study of the Italian network for research on psychoses. JAMA Psychiatry. (2021) 78:550–9. 10.1192/j.eurpsy.2021.135 33566071 PMC7876615

[28] Giuliani L Giordano GM Bucci P Pezzella P Brando F Galderisi S. Improving knowledge on pathways to functional outcome in schizophrenia: main results from the italian network for research on psychoses. Front Psychiatry. (2021) 12:791117. 10.3389/fpsyt.2021.791117 34970172 PMC8712575

[29] Galderisi S Kaiser S Bitter I Nordentoft M Mucci A Sabe M. EPA guidance on treatment of negative symptoms in schizophrenia. Eur Psychiatry. (2021) 64:e21. 10.1192/j.eurpsy.2021.13 33726883 PMC8057437

[30] Harvey PD. When does cognitive decline occur in the period prior to the first episode of schizophrenia? Psychiatry. (2009) 6:12–4. 19724764 PMC2728942

[31] Galderisi S Mucci A Bitter I Libiger J Bucci P Fleischhacker WW. Persistent negative symptoms in first episode patients with schizophrenia: results from the European First Episode Schizophrenia Trial. Eur Neuropsychopharmacol. (2013) 23:196–204. 10.1016/j.euroneuro.2012.04.019 22647933

[32] Austin SF Mors O Budtz-Jørgensen E Secher RG Hjorthøj CR Bertelsen M. Long-term trajectories of positive and negative symptoms in first episode psychosis: a 10year follow-up study in the OPUS cohort. Schizophr Res. (2015) 168:84–91. 10.1016/j.schres.2015.07.021 26265299

[33] Tripathi A Kar SK Shukla R. Cognitive deficits in schizophrenia: understanding the biological correlates and remediation strategies. Clin Psychopharmacol Neurosci. (2018) 16:7–17. 10.9758/cpn.2018.16.1.7 29397662 PMC5810454

[34] Harvey PD Isner EC. Cognition, social cognition, and functional capacity in early-onset schizophrenia. Child Adolesc Psychiatr Clin N Am. (2020) 29:171–82. 10.1016/j.chc.2019.08.008 31708046

[35] Vesterager L Christensen T Olsen BB Krarup G Melau M Forchhammer HB. Cognitive and clinical predictors of functional capacity in patients with first episode schizophrenia. Schizophr Res. (2012) 141:251–6. 10.1016/j.schres.2012.08.023 23017825

[36] Feldman R. What is resilience: an affiliative neuroscience approach. World Psychiatry. (2020) 19:132–50. 10.1002/wps.20729 32394561 PMC7215067

[37] Moura BM Isvoranu AM Kovacs V van Rooijen G van Amelsvoort T Simons CJP. The puzzle of functional recovery in schizophrenia-spectrum disorders-replicating a network analysis study. Schizophr Bull. (2022) 48:871–80. 10.1093/schbul/sbac018 35266000 PMC9212097

[38] Altamura A Fagiolini A Galderisi S Rocca P Rossi A. Integrated treatment of schizophrenia. (2015) 21:168–93.

[39] Lenroot R Bustillo JR Lauriello J Keith SJ. Integrated treatment of schizophrenia. Psychiatr Serv. (2003) 54:1499–507. 10.1176/appi.ps.54.11.1499 14600309

[40] Chien WT Leung SF Yeung FK Wong WK. Current approaches to treatments for schizophrenia spectrum disorders, part II: psychosocial interventions and patient-focused perspectives in psychiatric care. Neuropsychiatr Dis Treat. (2013) 9:1463–81. 10.2147/NDT.S49263 24109184 PMC3792827

[41] Chien WT Yip AL. Current approaches to treatments for schizophrenia spectrum disorders, part I: an overview and medical treatments. Neuropsychiatr Dis Treat. (2013) 9:1311–32. 10.2147/NDT.S37485 24049446 PMC3775702

[42] Acosta FJ Hernandez JL Pereira J Herrera J Rodriguez CJ. Medication adherence in schizophrenia. World J Psychiatry. (2012) 2:74–82. 10.5498/wjp.v2.i5.74 24175171 PMC3782179

[43] Lacro JP Dunn LB Dolder CR Leckband SG Jeste DV. Prevalence of and risk factors for medication nonadherence in patients with schizophrenia: a comprehensive review of recent literature. J Clin Psychiatry. (2002) 63:892–909. 10.4088/JCP.v63n1007 12416599

[44] Barnes TR. Evidence-based guidelines for the pharmacological treatment of schizophrenia: recommendations from the British Association for Psychopharmacology. J Psychopharmacol. (2011) 25:567–620. 10.1177/0269881110391123 21292923

[45] Morin L Franck N. Rehabilitation interventions to promote recovery from schizophrenia: a systematic review. Front Psychiatry. (2017) 8:100. 10.3389/fpsyt.2017.00100 28659832 PMC5467004

[46] Tandon R Targum SD Nasrallah HA Ross R. Strategies for maximizing clinical effectiveness in the treatment of schizophrenia. J Psychiatr Pract. (2006) 12:348–63. 10.1097/00131746-200611000-00003 17122696

[47] Kern RS Glynn SM Horan WP Marder SR. Psychosocial treatments to promote functional recovery in schizophrenia. Schizophr Bull. (2009) 35:347–61. 10.1093/schbul/sbn177 19176470 PMC2659313

[48] Barlati S Deste G Galluzzo A Perin AP Valsecchi P Turrina C. Factors associated with response and resistance to cognitive remediation in schizophrenia: a critical review. Front Pharmacol. (2018) 9:1542. 10.3389/fphar.2018.01542 30687100 PMC6335346

[49] Pharoah F Mari J Rathbone J Wong W. Family intervention for schizophrenia. Cochrane Database Syst Rev. (2010) 12:Cd000088. 10.1002/14651858.CD000088.pub3 21154340 PMC4204509

[50] National Collaborating Centre for Mental Health. National Institute for Health and Clinical Excellence: Guidance. Schizophrenia: Core Interventions in the Treatment and Management of Schizophrenia in Primary and Secondary Care (Update). Leicester: British Psychological Society Copyright (2009). 20704054

[51] Wykes T Steel C Everitt B Tarrier N. Cognitive behavior therapy for schizophrenia: effect sizes, clinical models, and methodological rigor. Schizophr Bull. (2008) 34:523–37. 10.1093/schbul/sbm114 17962231 PMC2632426

[52] Sarin F Wallin L. Cognitive model and cognitive behavior therapy for schizophrenia: an overview. Nord J Psychiatry. (2014) 68:145–53. 10.3109/08039488.2013.789074 24627960

[53] Nowak I Sabariego C Switaj P Anczewska M. Disability and recovery in schizophrenia: a systematic review of cognitive behavioral therapy interventions. BMC Psychiatry. (2016) 16:228. 10.1186/s12888-016-0912-8 27400680 PMC4940955

[54] Laws KR Darlington N Kondel TK McKenna PJ Jauhar S. Cognitive behavioural therapy for schizophrenia - outcomes for functioning, distress and quality of life: a meta-analysis. BMC Psychology. (2018) 6:32. 10.1186/s40359-018-0243-2 30016999 PMC6050679

[55] Wykes T. Cognitive remediation – where are we now and what should we do next? The Journal of Psychopathol (2018) 24:57–61.

[56] Cella M Price T Corboy H Onwumere J Shergill S Preti A. Cognitive remediation for inpatients with psychosis: a systematic review and meta-analysis. Psychol Med. (2020) 50:1062–76. 10.1017/S0033291720000872 32349802

[57] Kambeitz-Ilankovic L Betz LT Dominke C Haas SS Subramaniam K Fisher M. Multi-outcome meta-analysis (MOMA) of cognitive remediation in schizophrenia: revisiting the relevance of human coaching and elucidating interplay between multiple outcomes. Neurosci Biobehav Rev. (2019) 107:828–45. 10.1016/j.neubiorev.2019.09.031 31557548 PMC8942567

[58] Vita A Barlati S Ceraso A Deste G Nibbio G Wykes T. Acceptability of cognitive remediation for schizophrenia: a systematic review and meta-analysis of randomized controlled trials. Psychol Med. (2022) 1–11. 10.1017/S0033291722000319 PMC10277755 35257646

[59] Vita A Barlati S Ceraso A Nibbio G Ariu C Deste G. Effectiveness, core elements, and moderators of response of cognitive remediation for schizophrenia a systematic review and meta-analysis of randomized clinical trials. JAMA Psychiatry. (2021) 78:848–58. 10.1001/jamapsychiatry.2021.0620 33877289 PMC8058696

[60] Wykes T Huddy V Cellard C McGurk SR Czobor P. A meta-analysis of cognitive remediation for schizophrenia: methodology and effect sizes. Am J Psychiatry. (2011) 168:472–85. 10.1176/appi.ajp.2010.10060855 21406461

[61] Barlati S Deste G De Peri L Ariu C Vita A. Cognitive remediation in schizophrenia: current status and future perspectives. Schizophr Res Treatment. (2013) 2013:156084. 10.1155/2013/156084 24455253 PMC3877646

[62] Cella M Preti A Edwards C Dow T Wykes T. Cognitive remediation for negative symptoms of schizophrenia: a network meta-analysis. Clin Psychol Rev. (2017) 52:43–51. 10.1016/j.cpr.2016.11.009 27930934

[63] Cella M Stahl D Morris S Keefe RSE Bell MD Wykes T. Effects of cognitive remediation on negative symptoms dimensions: exploring the role of working memory. Psychol Med. (2017) 47:1–9. 10.1017/S0033291717000757 28866985 PMC5647678

[64] Lutgens D Gariepy G Malla A. Psychological and psychosocial interventions for negative symptoms in psychosis: systematic review and meta-analysis. Br J Psychiatry. (2017) 210:324–32. 10.1192/bjp.bp.116.197103 28302699

[65] Glenthøj LB Mariegaard LS Fagerlund B Jepsen JRM Kristensen TD Wenneberg C. Effectiveness of cognitive remediation in the ultra-high risk state for psychosis. World Psychiatry. (2020) 19:401–2. 10.1002/wps.20760 32931108 PMC7491627

[66] Turner DT McGlanaghy E Cuijpers P van der Gaag M Karyotaki E MacBeth A. A Meta-analysis of social skills training and related interventions for psychosis. Schizophr Bull. (2018) 44:475–91. 10.1093/schbul/sbx146 29140460 PMC5890475

[67] Browne J Mueser KT Pratt SI. Chapter 17 - social skills training for persons with schizophrenia. In: Nangle DW, Erdley CA, Schwartz-Mette RA, editors. Social Skills Across the Life Span. Academic Press (2020). p. 329–42.

[68] Thorup A Petersen L Jeppesen P Ohlenschlaeger J Christensen T Krarup G. Integrated treatment ameliorates negative symptoms in first episode psychosis–results from the Danish OPUS trial. Schizophr Res. (2005) 79:95–105. 10.1016/j.schres.2004.12.020 16122909

[69] Yeo H Yoon S Lee J Kurtz MM Choi K. A meta-analysis of the effects of social-cognitive training in schizophrenia: the role of treatment characteristics and study quality. Br J Clin Psychol. (2022) 61:37–57. 10.1111/bjc.12320 34291465

[70] Correll CU Galling B Pawar A Krivko A Bonetto C Ruggeri M. Comparison of early intervention services vs treatment as usual for early-phase psychosis: a systematic review, meta-analysis, and meta-regression. JAMA Psychiatry. (2018) 75:555–65. 10.1001/jamapsychiatry.2018.0623 29800949 PMC6137532

[71] Singh SP Javed A. Early intervention in psychosis in low- and middle-income countries: a WPA initiative. World Psychiatry. (2020) 19:122. 10.1002/wps.20708 31922694 PMC6953594

[72] Morrison AP Law H Carter L Sellers R Emsley R Pyle M. Antipsychotic drugs versus cognitive behavioural therapy versus a combination of both in people with psychosis: a randomised controlled pilot and feasibility study. Lancet Psychiatry. (2018) 5:411–23. 10.1016/S2215-0366(18)30096-8 29605187 PMC6048761

[73] Higashi K Medic G Littlewood KJ Diez T Granström O De Hert M. Medication adherence in schizophrenia: factors influencing adherence and consequences of nonadherence, a systematic literature review. Therap Adv Psychopharmacol. (2013) 3:200–18. 10.1177/2045125312474019 24167693 PMC3805432

[74] Fervaha G Agid O Takeuchi H Lee J Foussias G Zakzanis KK. Extrapyramidal symptoms and cognitive test performance in patients with schizophrenia. Schizophr Res. (2015) 161:351–6. 10.1016/j.schres.2014.11.018 25471015

[75] Monteleone P Cascino G Monteleone AM Rocca P Rossi A Bertolino A. Prevalence of antipsychotic-induced extrapyramidal symptoms and their association with neurocognition and social cognition in outpatients with schizophrenia in the “real-life”. Prog Neuropsychopharmacol Biol Psychiatry. (2021) 109:110250. 10.1016/j.pnpbp.2021.110250 33484755

[76] MacKenzie NE Kowalchuk C Agarwal SM Costa-Dookhan KA Caravaggio F Gerretsen P. Antipsychotics, metabolic adverse effects, and cognitive function in schizophrenia. Front Psychiatry. (2018) 9:622. 10.3389/fpsyt.2018.00622 30568606 PMC6290646

[77] Fond G Berna F Boyer L Godin O Brunel L Andrianarisoa M. Benzodiazepine long-term administration is associated with impaired attention/working memory in schizophrenia: results from the national multicentre FACE-SZ data set. Eur Arch Psychiatry Clin Neurosci. (2018) 268:17–26. 10.1007/s00406-017-0787-9 28349247

[78] Joshi YB Thomas ML Braff DL Green MF Gur RC Gur RE. Anticholinergic Medication Burden-Associated Cognitive Impairment in Schizophrenia. Am J Psychiatry. (2021) 178:838–47. 10.1176/appi.ajp.2020.20081212 33985348 PMC8440496

[79] Khan WU Ghazala Z Brooks HJ Subramaniam P Mulsant BH Kumar S. The impact of anticholinergic burden on functional capacity in persons with schizophrenia across the adult life span. Schizophr Bull. (2021) 47:249–57. 10.1093/schbul/sbaa093 32619225 PMC7825090

[80] Fleischhacker WW Oehl MA Hummer M. Factors influencing compliance in schizophrenia patients. J Clin Psychiatry. (2003) 64(Suppl. 16):10–3. 14680413

[81] Keshavan MS Eack S. Cognitive Enhancement in Schizophrenia and Related Disorders. Cambridge University Press (2019).

[82] Leucht S Tardy M Komossa K Heres S Kissling W Salanti G. Antipsychotic drugs versus placebo for relapse prevention in schizophrenia: a systematic review and meta-analysis. Lancet. (2012) 379:2063–71. 10.1016/S0140-6736(12)60239-6 22560607

[83] DeRosse P Nitzburg GC Blair M Malhotra AK. Dimensional symptom severity and global cognitive function predict subjective quality of life in patients with schizophrenia and healthy adults. Schizophr Res. (2018) 195:385–90. 10.1016/j.schres.2017.10.018 29056491 PMC5908765

[84] Cullen BA McGinty EE Zhang Y dosReis SC Steinwachs DM Guallar E. Guideline-concordant antipsychotic use and mortality in schizophrenia. Schizophr Bull. (2012) 39:1159–68. 10.1093/schbul/sbs097 23112292 PMC3756776

[85] Liu NH Daumit GL Dua T Aquila R Charlson F Cuijpers P. Excess mortality in persons with severe mental disorders: a multilevel intervention framework and priorities for clinical practice, policy and research agendas. World Psychiatry. (2017) 16:30–40. 10.1002/wps.20384 28127922 PMC5269481

[86] Miyamoto S Duncan GE Marx CE Lieberman JA. Treatments for schizophrenia: a critical review of pharmacology and mechanisms of action of antipsychotic drugs. Mol Psychiatry. (2005) 10:79–104. 10.1038/sj.mp.4001556 15289815

[87] Pringsheim T Addington D. Canadian schizophrenia guidelines: introduction and guideline development process. Can J Psychiatry. (2017) 62:586–93. 10.1177/0706743717719897 28789558 PMC5593245

[88] Pringsheim T Gardner D Addington D Martino D Morgante F Ricciardi L. The assessment and treatment of antipsychotic-induced Akathisia. Can J Psychiatry. (2018) 63:719–29. 10.1177/0706743718760288 29685069 PMC6299189

[89] Verdoux H Quiles C Bon L Chéreau-Boudet I Dubreucq J Legros-Lafarge E. Characteristics associated with self-reported medication adherence in persons with psychosis referred to psychosocial rehabilitation centers. Eur Arch Psychiatry Clin Neurosci. (2021) 271:1415–24. 10.1007/s00406-020-01207-x 33169212

[90] Taipale H Tanskanen A Mehtälä J Vattulainen P Correll CU Tiihonen J. 20-year follow-up study of physical morbidity and mortality in relationship to antipsychotic treatment in a nationwide cohort of 62,250 patients with schizophrenia (FIN20). World psychiatry. (2020) 19:61–8. 10.1002/wps.20699 31922669 PMC6953552

[91] Leong I. Side effects of olanzapine worsened by metabolic dysfunction. Nat Rev Endocrinol. (2018) 14:129. 10.1038/nrendo.2017.183 29286046

[92] Galderisi S De Hert M Del Prato S Fagiolini A Gorwood P Leucht S. Identification and management of cardiometabolic risk in subjects with schizophrenia spectrum disorders: a Delphi expert consensus study. Eur Psychiatry. (2021) 64:e7. 10.1192/j.eurpsy.2020.115 33413701 PMC8057390

[93] Sykes DA Moore H Stott L Holliday N Javitch JA Lane JR. Extrapyramidal side effects of antipsychotics are linked to their association kinetics at dopamine D(2) receptors. Nat Commun. (2017) 8:763. 10.1038/s41467-017-00716-z 28970469 PMC5624946

[94] Ames D Carr-Lopez SM Gutierrez MA Pierre JM Rosen JA Shakib S. Detecting and managing adverse effects of antipsychotic medications: current state of play. Psychiatr Clin North Am. (2016) 39:275–311. 10.1016/j.psc.2016.01.008 27216904

[95] Fabrazzo M Monteleone P Prisco V Perris F Catapano F Tortorella A. Olanzapine is faster than haloperidol in inducing metabolic abnormalities in schizophrenic and bipolar patients. Neuropsychobiology. (2015) 72:29–36. 10.1159/000437430 26337616

[96] Loonen AJ Ivanova SA. Neurobiological mechanisms associated with antipsychotic drug-induced dystonia. J Psychopharmacol. (2021) 35:3–14. 10.1177/0269881120944156 32900259 PMC7770213

[97] Misdrahi D Tessier A Daubigney A Meissner WG Schurhoff F Boyer L. Prevalence of and risk factors for extrapyramidal side effects of antipsychotics: results from the national FACE-SZ cohort. J Clin Psychiatry. (2019) 80:246. 10.4088/JCP.18m12246 30695288

[98] Yang SY Kao Yang YH Chong MY Yang YH Chang WH Lai CS. Risk of extrapyramidal syndrome in schizophrenic patients treated with antipsychotics: a population-based study. Clin Pharmacol Ther. (2007) 81:586–94. 10.1038/sj.clpt.6100069 17235331

[99] Rummel-Kluge C Komossa K Schwarz S Hunger H Schmid F Kissling W. Second-generation antipsychotic drugs and extrapyramidal side effects: a systematic review and meta-analysis of head-to-head comparisons. Schizophr Bull. (2012) 38:167–77. 10.1093/schbul/sbq042 20513652 PMC3245581

[100] Leucht S Cipriani A Spineli L Mavridis D Orey D Richter F. Comparative efficacy and tolerability of 15 antipsychotic drugs in schizophrenia: a multiple-treatments meta-analysis. Lancet. (2013) 382:951–62. 10.1016/S0140-6736(13)60733-3 23810019

[101] Solmi M Murru A Pacchiarotti I Undurraga J Veronese N Fornaro M. Safety, tolerability, and risks associated with first- and second-generation antipsychotics: a state-of-the-art clinical review. Ther Clin Risk Manag. (2017) 13:757–77. 10.2147/TCRM.S117321 28721057 PMC5499790

[102] Galderisi S Mucci A Dollfus S Nordentoft M Falkai P Kaiser S. EPA guidance on assessment of negative symptoms in schizophrenia. Eur Psychiatry. (2021) 64:e23. 10.1192/j.eurpsy.2021.11 33597064 PMC8080207

[103] Galderisi S Mucci A Buchanan RW Arango C. Negative symptoms of schizophrenia: new developments and unanswered research questions. Lancet Psychiatry. (2018) 5:664–77. 10.1016/S2215-0366(18)30050-6 29602739

[104] Strachan MW Deary IJ Ewing FM Frier BM. Is type II diabetes associated with an increased risk of cognitive dysfunction? A critical review of published studies. Diabetes Care. (1997) 20:438–45. 10.2337/diacare.20.3.438 9051402

[105] Ryan JD Althoff RR Whitlow S Cohen NJ. Amnesia is a deficit in relational memory. Psychol Sci. (2000) 11:454–61. 10.1111/1467-9280.00288 11202489

[106] Ryan JD Cohen NJ. Processing and short-term retention of relational information in amnesia. Neuropsychologia. (2004) 42:497–511. 10.1016/j.neuropsychologia.2003.08.011 14728922

[107] Pavlik VN Hyman DJ Doody R. Cardiovascular risk factors and cognitive function in adults 30-59 years of age (NHANES III). Neuroepidemiology. (2005) 24:42–50. 10.1159/000081049 15459509

[108] Bruehl H Rueger M Dziobek I Sweat V Tirsi A Javier E. Hypothalamic-pituitary-adrenal axis dysregulation and memory impairments in type 2 diabetes. J Clin Endocrinol Metab. (2007) 92:2439–45. 10.1210/jc.2006-2540 17426095

[109] Lindenmayer JP Khan A Kaushik S Thanju A Praveen R Hoffman L. Relationship between metabolic syndrome and cognition in patients with schizophrenia. Schizophr Res. (2012) 142:171–6. 10.1016/j.schres.2012.09.019 23106932

[110] Li C Zhan G Rao S Zhang H. Metabolic syndrome and its factors affect cognitive function in chronic schizophrenia complicated by metabolic syndrome. J Nerv Ment Dis. (2014) 202:313–8. 10.1097/NMD.0000000000000124 24647221

[111] Bora E Akdede BB Alptekin K. The relationship between cognitive impairment in schizophrenia and metabolic syndrome: a systematic review and meta-analysis. Psychol Med. (2017) 47:1030–40. 10.1017/S0033291716003366 28032535

[112] Papola D Ostuzzi G Gastaldon C Morgano GP Dragioti E Carvalho AF. Antipsychotic use and risk of life-threatening medical events: umbrella review of observational studies. Acta Psychiatr Scand. (2019) 140:227–43. 10.1111/acps.13066 31264708

[113] Stroup TS Gray N. Management of common adverse effects of antipsychotic medications. World Psychiatry. (2018) 17:341–56. 10.1002/wps.20567 30192094 PMC6127750

[114] Gupta S Lakshmanan DAM Khastgir U Nair R. Management of antipsychotic-induced hyperprolactinaemia. BJPsych Advances. (2017) 23:278–86. 10.1192/apt.bp.115.014928

[115] Fabrazzo M Prisco V Sampogna G Perris F Catapano F Monteleone A. Clozapine versus other antipsychotics during the first 18 weeks of treatment: a retrospective study on risk factor increase of blood dyscrasias. Psychiatry Res. (2017) 256:275–82. 10.1016/j.psychres.2017.06.068 28651220

[116] Velligan DI Weiden PJ Sajatovic M Scott J Carpenter D Ross R. The expert consensus guideline series: adherence problems in patients with serious and persistent mental illness. J Clin Psychiatry. (2009) 70(Suppl. 4):1–46; quiz 7–8. 10.4088/JCP.7090su1cj 19686636

[117] Tsoutsoulas C Mulsant BH Kalache SM Kumar S Ghazala Z Voineskos AN. The influence of medical burden severity and cognition on functional competence in older community-dwelling individuals with schizophrenia. Schizophr Res. (2016) 170:330–5. 10.1016/j.schres.2015.12.009 26724896

[118] Baandrup L Fagerlund B Glenthoj B. Neurocognitive performance, subjective well-being, and psychosocial functioning after benzodiazepine withdrawal in patients with schizophrenia or bipolar disorder: a randomized clinical trial of add-on melatonin versus placebo. Eur Arch Psychiatry Clin Neurosci. (2017) 267:163–71. 10.1007/s00406-016-0711-8 27400927

[119] de la Iglesia-Larrad JI Barral C Casado-Espada NM de Alarcón R Maciá-Casas A Vicente Hernandez B. Benzodiazepine abuse, misuse, dependence, and withdrawal among schizophrenic patients: a review of the literature. Psychiatry Res. (2020) 284:112660. 10.1016/j.psychres.2019.112660 31757643

[120] Stewart SA. The effects of benzodiazepines on cognition. J Clin Psychiatry. (2005) 66(Suppl. 2):9–13. 15762814

[121] Verdoux H Quiles C Bon L Chereau-Boudet I Dubreucq J Fiegi L. Impact of anticholinergic load on functioning and cognitive performances of persons with psychosis referred to psychosocial rehabilitation centers. Psychol Med. (2020) 51:1–9. 10.1017/S0033291720001403 32441236

[122] Ogino S Miyamoto S Miyake N Yamaguchi N. Benefits and limits of anticholinergic use in schizophrenia: focusing on its effect on cognitive function. Psychiatry Clin Neurosci. (2014) 68:37–49. 10.1111/pcn.12088 24102938

[123] O'Reilly K O'Connell P Donohoe G Coyle C O'Sullivan D Azvee Z. Anticholinergic burden in schizophrenia and ability to benefit from psychosocial treatment programmes: a 3-year prospective cohort study. Psychol Med. (2016) 46:3199–211. 10.1017/S0033291716002154 27576609

[124] Kim SJ Jung D Shim JC Moon JJ Jeon DW Kim YN. The effect of anticholinergic burden on cognitive and daily living functions in patients with schizophrenia. Asian J Psychiatr. (2019) 46:111–7. 10.1016/j.ajp.2019.10.013 31654923

[125] Ang MS Abdul Rashid NA Lam M Rapisarda A Kraus M Keefe RSE. The impact of medication anticholinergic burden on cognitive performance in people with schizophrenia. J Clin Psychopharmacol. (2017) 37:651–6. 10.1097/JCP.0000000000000790 29016375 PMC5680994

[126] Frydecka D Beszłej JA Gościmski P Kiejna A Misiak B. Profiling cognitive impairment in treatment-resistant schizophrenia patients. Psychiatry Res. (2016) 235:133–8. 10.1016/j.psychres.2015.11.028 26706131

[127] Eum S Hill SK Rubin LH Carnahan RM Reilly JL Ivleva EI. Cognitive burden of anticholinergic medications in psychotic disorders. Schizophr Res. (2017) 190:129–35. 10.1016/j.schres.2017.03.034 28390849 PMC5628100

[128] Fond G Godin O Dumontaud M Faget C Schürhoff F Berna F. Sexual dysfunctions are associated with major depression, chronic inflammation and anticholinergic consumption in the real-world schizophrenia FACE-SZ national cohort. Prog Neuro-Psychopharmacol Biol Psychiatry. (2019) 94:109654. 10.1016/j.pnpbp.2019.109654 31125587

[129] Desmarais JE Beauclair L Annable L Bélanger MC Kolivakis TT Margolese HC. Effects of discontinuing anticholinergic treatment on movement disorders, cognition and psychopathology in patients with schizophrenia. Ther Adv Psychopharmacol. (2014) 4:257–67. 10.1177/2045125314553611 25489477 PMC4257986

[130] Kane JM. Treatment adherence and long-term outcomes. CNS Spectr. (2007) 12(Suppl. 17):21–6. 10.1017/S1092852900026304 17934386

[131] Morken G Widen JH Grawe RW. Non-adherence to antipsychotic medication, relapse and rehospitalisation in recent-onset schizophrenia. BMC Psychiatry. (2008) 8:32. 10.1186/1471-244X-8-32 18447935 PMC2390550

[132] Weiden PJ. Understanding and addressing adherence issues in schizophrenia: from theory to practice. J Clin Psychiatry. (2007) 68(Suppl. 14):14–9. 18284273

[133] Kane JM Garcia-Ribera C. Clinical guideline recommendations for antipsychotic long-acting injections. Br J Psychiatry Suppl. (2009) 52:S63–7. 10.1192/bjp.195.52.s63 19880920

[134] Amore M Murri MB Calcagno P Rocca P Rossi A Aguglia E. The association between insight and depressive symptoms in schizophrenia: undirected and Bayesian network analyses. European Psychiatry. (2020) 63:e46. 10.1192/j.eurpsy.2020.45 32372731 PMC7358633

[135] Beck EM Cavelti M Kvrgic S Kleim B Vauth R. Are we addressing the 'right stuff' to enhance adherence in schizophrenia? Understanding the role of insight and attitudes towards medication. Schizophr Res. (2011) 132:42–9. 10.1016/j.schres.2011.07.019 21820875

[136] Kao YC Liu YP. Compliance and schizophrenia: the predictive potential of insight into illness, symptoms, and side effects. Compr Psychiatry. (2010) 51:557–65. 10.1016/j.comppsych.2010.03.007 20965300

[137] Velligan DI Sajatovic M Hatch A Kramata P Docherty JP. Why do psychiatric patients stop antipsychotic medication? A systematic review of reasons for nonadherence to medication in patients with serious mental illness. Patient Prefer Adherence. (2017) 11:449–68. 10.2147/PPA.S124658 28424542 PMC5344423

[138] Fenton WS Blyler CR Heinssen RK. Determinants of medication compliance in schizophrenia: empirical and clinical findings. Schizophr Bull. (1997) 23:637–51. 10.1093/schbul/23.4.637 9366000

[139] Mohamed S Rosenheck R McEvoy J Swartz M Stroup S Lieberman JA. Cross-sectional and longitudinal relationships between insight and attitudes toward medication and clinical outcomes in chronic schizophrenia. Schizophr Bull. (2009) 35:336–46. 10.1093/schbul/sbn067 18586692 PMC2659303

[140] Lindenmayer JP Liu-Seifert H Kulkarni PM Kinon BJ Stauffer V Edwards SE. Medication nonadherence and treatment outcome in patients with schizophrenia or schizoaffective disorder with suboptimal prior response. J Clin Psychiatry. (2009) 70:990–6. 10.4088/JCP.08m04221 19497244

[141] Donohoe G Owens N O'Donnell C Burke T Moore L Tobin A. Predictors of compliance with neuroleptic medication among inpatients with schizophrenia: a discriminant function analysis. Eur Psychiatry. (2001) 16:293–8. 10.1016/S0924-9338(01)00581-8 11514132

[142] Robinson DG Woerner MG Alvir JM Bilder RM Hinrichsen GA Lieberman JA. Predictors of medication discontinuation by patients with first-episode schizophrenia and schizoaffective disorder. Schizophr Res. (2002) 57:209–19. 10.1016/S0920-9964(01)00312-7 12223252

[143] Jeste SD Patterson TL Palmer BW Dolder CR Goldman S Jeste DV. Cognitive predictors of medication adherence among middle-aged and older outpatients with schizophrenia. Schizophr Res. (2003) 63:49–58. 10.1016/S0920-9964(02)00314-6 12892857

[144] Keith SJ Kane JM. Partial compliance and patient consequences in schizophrenia: our patients can do better. J Clin Psychiatry. (2003) 64:1308–15. 10.4088/JCP.v64n1105 14658944

[145] Adams SG Jr. Howe JT. Predicting medication compliance in a psychotic population. J Nerv Ment Dis. (1993) 181:558–60. 10.1097/00005053-199309000-00005 7902413

[146] Buchanan A. A two-year prospective study of treatment compliance in patients with schizophrenia. Psychol Med. (1992) 22:787–97. 10.1017/S0033291700038228 1357703

[147] Kemp R David A. Psychological predictors of insight and compliance in psychotic patients. Br J Psychiatry. (1996) 169:444–50. 10.1192/bjp.169.4.444 8894195

[148] Yang J Ko YH Paik JW Lee MS Han C Joe SH. Symptom severity and attitudes toward medication: impacts on adherence in outpatients with schizophrenia. Schizophr Res. (2012) 134:226–31. 10.1016/j.schres.2011.11.008 22133906

[149] Jónsdóttir H Opjordsmoen S Birkenaes AB Simonsen C Engh JA Ringen PA. Predictors of medication adherence in patients with schizophrenia and bipolar disorder. Acta Psychiatr Scand. (2013) 127:23–33. 10.1111/j.1600-0447.2012.01911.x 22900964

[150] Klingberg S Schneider S Wittorf A Buchkremer G Wiedemann G. Collaboration in outpatient antipsychotic drug treatment: analysis of potentially influencing factors. Psychiatry Res. (2008) 161:225–34. 10.1016/j.psychres.2007.07.027 18922582

[151] Lepage M Bodnar M Joober R Malla A. Is there an association between neurocognitive performance and medication adherence in first episode psychosis? Early Interv Psychiatry. (2010) 4:189–95. 10.1111/j.1751-7893.2010.00174.x 20536976

[152] Perkins DO Gu H Weiden PJ McEvoy JP Hamer RM Lieberman JA. Predictors of treatment discontinuation and medication nonadherence in patients recovering from a first episode of schizophrenia, schizophreniform disorder, or schizoaffective disorder: a randomized, double-blind, flexible-dose, multicenter study. J Clin Psychiatry. (2008) 69:106–13. 10.4088/JCP.v69n0114 18312044

[153] Hudson TJ Owen RR Thrush CR Han X Pyne JM Thapa P. A pilot study of barriers to medication adherence in schizophrenia. J Clin Psychiatry. (2004) 65:211–6. 10.4088/JCP.v65n0211 15003075

[154] Verdoux H Lengronne J Liraud F Gonzales B Assens F Abalan F. Medication adherence in psychosis: predictors and impact on outcome. A 2-year follow-up of first-admitted subjects. Acta Psychiatr Scand. (2000) 102:203–10. 10.1034/j.1600-0447.2000.102003203.x 11008856

[155] Tattan TM Creed FH. Negative symptoms of schizophrenia and compliance with medication. Schizophr Bull. (2001) 27:149–55. 10.1093/oxfordjournals.schbul.a006853 11215543

[156] Rettenbacher MA Hofer A Eder U Hummer M Kemmler G Weiss EM. Compliance in schizophrenia: psychopathology, side effects, and patients' attitudes toward the illness and medication. J Clin Psychiatry. (2004) 65:1211–8. 10.4088/JCP.v65n0908 15367047

[157] Linden M Godemann F Gaebel W Köpke W Müller P Müller-Spahn F. A prospective study of factors influencing adherence to a continuous neuroleptic treatment program in schizophrenia patients during 2 years. Schizophr Bull. (2001) 27:585–96. 10.1093/oxfordjournals.schbul.a006898 11824485

[158] Goff DC Hill M Freudenreich O. Strategies for improving treatment adherence in schizophrenia and schizoaffective disorder. J Clin Psychiatry. (2010) 71(Suppl. 2):20–6. 10.4088/JCP.9096su1cc.04 21190649

[159] Hasan A Falkai P Wobrock T Lieberman J Glenthoj B Gattaz WF. World Federation of Societies of Biological Psychiatry (WFSBP) Guidelines for Biological Treatment of Schizophrenia, part 1: update 2012 on the acute treatment of schizophrenia and the management of treatment resistance. World J Biol Psychiatry. (2012) 13:318–78. 10.3109/15622975.2012.696143 22834451

[160] Leporini C De Sarro G Russo E. Adherence to therapy and adverse drug reactions: is there a link? Expert Opin Drug Saf. (2014) 13(Suppl. 1):S41–55. 10.1517/14740338.2014.947260 25171158

[161] Marcum ZA Gellad WF. Medication adherence to multidrug regimens. Clin Geriatr Med. (2012) 28:287–300. 10.1016/j.cger.2012.01.008 22500544 PMC3335752

[162] Zelko E Klemenc-Ketis Z Tusek-Bunc K. Medication adherence in elderly with polypharmacy living at home. a systematic review of existing studies. Mater Sociomed. (2016) 28:129–32. 10.5455/msm.2016.28.129-132 27147920 PMC4851507

[163] Kreyenbuhl JA Valenstein M McCarthy JF Ganoczy D Blow FC. Long-term antipsychotic polypharmacy in the VA health system: patient characteristics and treatment patterns. Psychiatr Serv. (2007) 58:489–95. 10.1176/ps.2007.58.4.489 17412850 PMC3673552

[164] Barbui C Biancosino B Esposito E Marmai L Donà S Grassi L. Factors associated with antipsychotic dosing in psychiatric inpatients: a prospective study. Int Clin Psychopharmacol. (2007) 22:221–5. 10.1097/YIC.0b013e3281084ea8 17519645

[165] Centorrino F Goren JL Hennen J Salvatore P Kelleher JP Baldessarini RJ. Multiple versus single antipsychotic agents for hospitalized psychiatric patients: case-control study of risks versus benefits. Am J Psychiatry. (2004) 161:700–6. 10.1176/appi.ajp.161.4.700 15056517

[166] Kreyenbuhl J Valenstein M McCarthy JF Ganoczy D Blow FC. Long-term combination antipsychotic treatment in VA patients with schizophrenia. Schizophr Res. (2006) 84:90–9. 10.1016/j.schres.2006.02.023 16631354

[167] Lähteenvuo M Tiihonen J. Antipsychotic polypharmacy for the management of schizophrenia: evidence and recommendations. Drugs. (2021) 81:1273–84. 10.1007/s40265-021-01556-4 34196945 PMC8318953

[168] Diaz E Neuse E Sullivan MC Pearsall HR Woods SW. Adherence to conventional and atypical antipsychotics after hospital discharge. J Clin Psychiatry. (2004) 65:354–60. 10.4088/JCP.v65n0311 15096075

[169] Burton SC. Strategies for improving adherence to second-generation antipsychotics in patients with schizophrenia by increasing ease of use. J Psychiatr Pract. (2005) 11:369–78. 10.1097/00131746-200511000-00003 16304505

[170] Remington G Kwon J Collins A Laporte D Mann S Christensen B. The use of electronic monitoring (MEMS) to evaluate antipsychotic compliance in outpatients with schizophrenia. Schizophr Res. (2007) 90:229–37. 10.1016/j.schres.2006.11.015 17208414

[171] Tareke M Tesfaye S Amare D Belete T Abate A. Antipsychotic medication non-adherence among schizophrenia patients in Central Ethiopia. South Afr J Psychiatry. (2018). 24:1124. 10.4102/sajpsychiatry.v24i0.1124 PMC6138084 30263211

[172] Hashimoto Y Uno J Miwa T Kurihara M Tanifuji H Tensho M. Effects of antipsychotic polypharmacy on side-effects and concurrent use of medications in schizophrenic outpatients. Psychiatry Clin Neurosci. (2012) 66:405–10. 10.1111/j.1440-1819.2012.02376.x 22834658

[173] Chapman SC Horne R. Medication nonadherence and psychiatry. Curr Opin Psychiatry. (2013) 26:446–52. 10.1097/YCO.0b013e3283642da4 23880592 PMC4222796

[174] Ishii M Okumura Y Sugiyama N Hasegawa H Noda T Hirayasu Y. Efficacy of shared decision making on treatment satisfaction for patients with first-admission schizophrenia: study protocol for a randomised controlled trial. BMC Psychiatry. (2014) 14:111. 10.1186/1471-244X-14-111 24725910 PMC4021257

[175] Fiorillo A Barlati S Bellomo A Corrivetti G Nicolò G Sampogna G. The role of shared decision-making in improving adherence to pharmacological treatments in patients with schizophrenia: a clinical review. Ann Gen Psychiatry. (2020) 19:43. 10.1186/s12991-020-00293-4 32774442 PMC7409631

[176] Tibaldi G Salvador-Carulla L García-Gutierrez JC. From treatment adherence to advanced shared decision making: new professional strategies and attitudes in mental health care. Curr Clin Pharmacol. (2011) 6:91–9. 10.2174/157488411796151101 21592062

[177] Ishii M Okumura Y Sugiyama N Hasegawa H Noda T Hirayasu Y. Feasibility and efficacy of shared decision making for first-admission schizophrenia: a randomized clinical trial. BMC Psychiatry. (2017) 17:52. 10.1186/s12888-017-1218-1 28166757 PMC5294770

[178] Mucci A Kawohl W Maria C Wooller A. Treating schizophrenia: open conversations and stronger relationships through psychoeducation and shared decision-making. Front Psychiatry. (2020) 11:761. 10.3389/fpsyt.2020.00761 32903708 PMC7438851

[179] Sendt KV Tracy DK Bhattacharyya S. A systematic review of factors influencing adherence to antipsychotic medication in schizophrenia-spectrum disorders. Psychiatry Res. (2015) 225:14–30. 10.1016/j.psychres.2014.11.002 25466227

[180] Huang C Lam L Zhong Y Plummer V Cross W. Chinese mental health professionals' perceptions of shared decision-making regarding people diagnosed with schizophrenia: a qualitative study. Int J Ment Health Nurs. (2021) 30:189–99. 10.1111/inm.12771_1 33300252

[181] Vega D Acosta FJ Saavedra P. Testing the hypothesis of subtypes of nonadherence in schizophrenia and schizoaffective disorder: a prospective study. World J Psychiatry. (2020) 10:260–71. 10.5498/wjp.v10.i11.260 33269222 PMC7672786

[182] Harris BA Panozzo G. Therapeutic alliance, relationship building, and communication strategies-for the schizophrenia population: an integrative review. Arch Psychiatr Nurs. (2019) 33:104–11. 10.1016/j.apnu.2018.08.003 30663612

[183] Johansen R Iversen VC Melle I Hestad KA. Therapeutic alliance in early schizophrenia spectrum disorders: a cross-sectional study. Ann Gen Psychiatry. (2013) 12:14. 10.1186/1744-859X-12-14 23656747 PMC3661357

[184] Löffler W Kilian R Toumi M Angermeyer MC. Schizophrenic patients' subjective reasons for compliance and noncompliance with neuroleptic treatment. Pharmacopsychiatry. (2003) 36:105–12. 10.1055/s-2003-39985 12806568

[185] Day JC Bentall RP Roberts C Randall F Rogers A Cattell D. Attitudes toward antipsychotic medication: the impact of clinical variables and relationships with health professionals. Arch Gen Psychiatry. (2005) 62:717–24. 10.1001/archpsyc.62.7.717 15997012

[186] National Institute for Health and Clinical. Medicines Adherence: Involving Patients in Decisions About Prescribed Medicines and Supporting Adherence. Clinical Guideline Clinical guidelines, CG76. London: NICE (2009). 39480983

[187] Green MF Nuechterlein KH Gold JM Barch DM Cohen J Essock S. Approaching a consensus cognitive battery for clinical trials in schizophrenia: the NIMH-MATRICS conference to select cognitive domains and test criteria. Biol Psychiatry. (2004) 56:301–7. 10.1016/j.biopsych.2004.06.023 15336511

[188] Nuechterlein KH Green MF Kern RS Baade LE Barch DM Cohen JD. The MATRICS Consensus Cognitive Battery, part 1: test selection, reliability, and validity. Am J Psychiatry. (2008) 165:203–13. 10.1176/appi.ajp.2007.07010042 18172019

[189] Green MF Penn DL Bentall R Carpenter WT Gaebel W Gur RC. Social cognition in schizophrenia: an NIMH workshop on definitions, assessment, and research opportunities. Schizophr Bull. (2008) 34:1211–20. 10.1093/schbul/sbm145 18184635 PMC2632490

[190] Green MF Lee J Wynn JK. Experimental approaches to social disconnection in the general community: can we learn from schizophrenia research? World Psychiatry. (2020) 19:177–8. 10.1002/wps.20734 32394575 PMC7215060

[191] Lysaker PH Hasson-Ohayon I. Metacognition in psychosis: a renewed path to understanding of core disturbances and recovery-oriented treatment. World Psychiatry. (2021) 20:359–61. 10.1002/wps.20914 34505380 PMC8429316

[192] Heckers S Kendler KS. The evolution of Kraepelin's nosological principles. World Psychiatry. (2020) 19:381–8. 10.1002/wps.20774 32931122 PMC7491624

[193] Giordano GM Palumbo D Mucci A Ventura J Giuliani L Perrottelli A. The Cognitive Assessment Interview (CAI): association with neuropsychological scores and real-life functioning in a large sample of Italian subjects with schizophrenia. Schizophr Res. (2022) 241:161–70. 10.1016/j.schres.2022.01.029 35124435

[194] Nielsen RE Levander S Kjaersdam Telléus G Jensen SO Østergaard Christensen T Leucht S. Second-generation antipsychotic effect on cognition in patients with schizophrenia–a meta-analysis of randomized clinical trials. Acta Psychiatr Scand. (2015) 131:185–96. 10.1111/acps.12374 25597383

[195] Dauwan M Begemann MJH Slot MIE Lee EHM Scheltens P Sommer IEC. Physical exercise improves quality of life, depressive symptoms, and cognition across chronic brain disorders: a transdiagnostic systematic review and meta-analysis of randomized controlled trials. J Neurol. (2021) 268:1222–46. 10.1007/s00415-019-09493-9 31414194 PMC7990819

[196] Fernández-Abascal B Suárez-Pinilla P Cobo-Corrales C Crespo-Facorro B Suárez-Pinilla M. In- and outpatient lifestyle interventions on diet and exercise and their effect on physical and psychological health: a systematic review and meta-analysis of randomised controlled trials in patients with schizophrenia spectrum disorders and first episode of psychosis. Neurosci Biobehav Rev. (2021) 125:535–68. 10.1016/j.neubiorev.2021.01.005 33503476

[197] Firth J Stubbs B Rosenbaum S Vancampfort D Malchow B Schuch F. Aerobic exercise improves cognitive functioning in people with schizophrenia: a systematic review and meta-analysis. Schizophr Bull. (2017) 43:546–56. 10.1093/schbul/sbw115 27521348 PMC5464163

[198] Li J Shen J Wu G Tan Y Sun Y Keller E. Mindful exercise versus non-mindful exercise for schizophrenia: a systematic review and meta-analysis of randomized controlled trials. Complement Ther Clin Pract. (2018) 32:17–24. 10.1016/j.ctcp.2018.04.003 30057047

[199] Millman LSM Terhune DB Hunter ECM Orgs G. Towards a neurocognitive approach to dance movement therapy for mental health: a systematic review. Clin Psychol Psychother. (2021) 28:24–38. 10.1002/cpp.2490 32539160

[200] Sin J Gillard S Spain D Cornelius V Chen T Henderson C. Effectiveness of psychoeducational interventions for family carers of people with psychosis: a systematic review and meta-analysis. Clin Psychol Rev. (2017) 56:13–24. 10.1016/j.cpr.2017.05.002 28578249

[201] Lincoln TM Wilhelm K Nestoriuc Y. Effectiveness of psychoeducation for relapse, symptoms, knowledge, adherence and functioning in psychotic disorders: a meta-analysis. Schizophr Res. (2007) 96:232–45. 10.1016/j.schres.2007.07.022 17826034

[202] Menon V. Brain networks and cognitive impairment in psychiatric disorders. World Psychiatry. (2020) 19:309–10. 10.1002/wps.20799 32931097 PMC7491636

[203] Moritz S Silverstein SM Dietrichkeit M Gallinat J. Neurocognitive deficits in schizophrenia are likely to be less severe and less related to the disorder than previously thought. World Psychiatry. (2020) 19:254–5. 10.1002/wps.20759 32394552 PMC7215075

[204] Reser MP Slikboer R Rossell SL. A systematic review of factors that influence the efficacy of cognitive remediation therapy in schizophrenia. Aust N Z J Psychiatry. (2019) 53:624–41. 10.1177/0004867419853348 31177813

[205] Vinogradov S Fisher M de Villers-Sidani E. Cognitive training for impaired neural systems in neuropsychiatric illness. Neuropsychopharmacology. (2012) 37:43–76. 10.1038/npp.2011.251 22048465 PMC3238091

[206] Gabay AS Kempton MJ Mehta MA. Facial affect processing deficits in schizophrenia: a meta-analysis of antipsychotic treatment effects. J Psychopharmacol. (2015) 29:224–9. 10.1177/0269881114560184 25492885 PMC4361469

[207] Thornton AE Van Snellenberg JX Sepehry AA Honer W. The impact of atypical antipsychotic medications on long-term memory dysfunction in schizophrenia spectrum disorder: a quantitative review. J Psychopharmacol. (2006) 20:335–46. 10.1177/0269881105057002 16174678

[208] Woodward ND Purdon SE Meltzer HY Zald DH. A meta-analysis of neuropsychological change to clozapine, olanzapine, quetiapine, and risperidone in schizophrenia. Int J Neuropsychopharmacol. (2005) 8:457–72. 10.1017/S146114570500516X 15784157

[209] Zhang JP Gallego JA Robinson DG Malhotra AK Kane JM Correll CU. Efficacy and safety of individual second-generation vs. first-generation antipsychotics in first-episode psychosis: a systematic review and meta-analysis. Int J Neuropsychopharmacol. (2013) 16:1205–18. 10.1017/S1461145712001277 23199972 PMC3594563

[210] Baldez DP Biazus TB Rabelo-da-Ponte FD Nogaro GP Martins DS Kunz M. The effect of antipsychotics on the cognitive performance of individuals with psychotic disorders: network meta-analyses of randomized controlled trials. Neurosci Biobehav Rev. (2021) 126:265–75. 10.1016/j.neubiorev.2021.03.028 33812977

[211] Keefe RS Bilder RM Davis SM Harvey PD Palmer BW Gold JM. Neurocognitive effects of antipsychotic medications in patients with chronic schizophrenia in the CATIE Trial. Arch Gen Psychiatry. (2007) 64:633–47. 10.1001/archpsyc.64.6.633 17548746

[212] Vita A Mussoni C Deste G Ferlenghi G Turrina C Valsecchi P. Psychopharmacological treatment of cognitive deficits in Schizophrenia and mood disorders. Off J Ital Soc Psychopathol. (2018) 24:62–72.

[213] Rodewald K Holt DV Rentrop M Roesch-Ely D Liebrenz M Funke J. Predictors for improvement of problem-solving during cognitive remediation for patients with Schizophrenia. J Int Neuropsychol Soc. (2014) 20:455–60. 10.1017/S1355617714000162 24589198

[214] Vita A Deste G De Peri L Barlati S Poli R Cesana BM. Predictors of cognitive and functional improvement and normalization after cognitive remediation in patients with schizophrenia. Schizophr Res. (2013) 150:51–7. 10.1016/j.schres.2013.08.011 23998953

[215] Tanaka T Tomotake M Ueoka Y Kaneda Y Taniguchi K Nakataki M. Clinical correlates associated with cognitive dysfunction in people with schizophrenia. Psychiatry Clin Neurosci. (2012) 66:491–8. 10.1111/j.1440-1819.2012.02390.x 23066766

[216] Potvin S Aubin G Stip E. Antipsychotic-induced parkinsonism is associated with working memory deficits in schizophrenia-spectrum disorders. Eur Arch Psychiatry Clin Neurosci. (2015) 265:147–54. 10.1007/s00406-014-0511-y 24925606

[217] Palmer BW Heaton RK Jeste DV. Extrapyramidal symptoms and neuropsychological deficits in schizophrenia. Biol Psychiatry. (1999) 45:791–4. 10.1016/S0006-3223(98)00167-X 10188011

[218] Kasper S Resinger E. Cognitive effects and antipsychotic treatment. Psychoneuroendocrinology. (2003) 28:27–38. 10.1016/S0306-4530(02)00115-4 12504070

[219] Cuesta MJ Sánchez-Torres AM de Jalón EG Campos MS Ibáñez B Moreno-Izco L. Spontaneous Parkinsonism is associated with cognitive impairment in antipsychotic-naive patients with first-episode psychosis: a 6-month follow-up study. Schizophr Bull. (2013) 40:1164–73. 10.1093/schbul/sbt125 24072809 PMC4133659

[220] Pantelis C Stuart GW Nelson HE Robbins TW Barnes TR. Spatial working memory deficits in schizophrenia: relationship with tardive dyskinesia and negative symptoms. Am J Psychiatry. (2001) 158:1276–85. 10.1176/appi.ajp.158.8.1276 11481163

[221] Wu JQ Chen DC Xiu MH Tan YL Yang FD Kosten TR. Tardive dyskinesia is associated with greater cognitive impairment in schizophrenia. Prog Neuropsychopharmacol Biol Psychiatry. (2013) 46:71–7. 10.1016/j.pnpbp.2013.06.013 23827756

[222] Miller DD McEvoy JP Davis SM Caroff SN Saltz BL Chakos MH. Clinical correlates of tardive dyskinesia in schizophrenia: baseline data from the CATIE schizophrenia trial. Schizophr Res. (2005) 80:33–43. 10.1016/j.schres.2005.07.034 16171976

[223] Waddington JL Ocallaghan E Larkin C Kinsella A. Cognitive dysfunction in schizophrenia: organic vulnerability factor or state marker for tardive dyskinesia? Brain Cogn. (1993) 23:56–70. 10.1006/brcg.1993.1044 8105822

[224] De Hert M Schreurs V Vancampfort D Van Winkel R. Metabolic syndrome in people with schizophrenia: a review. World Psychiatry. (2009) 8:15–22. 10.1002/j.2051-5545.2009.tb00199.x 19293950 PMC2656262

[225] Meyer JM Nasrallah HA McEvoy JP Goff DC Davis SM Chakos M. The clinical antipsychotic trials of intervention effectiveness (CATIE) schizophrenia trial: clinical comparison of subgroups with and without the metabolic syndrome. Schizophr Res. (2005) 80:9–18. 10.1016/j.schres.2005.07.015 16125372

[226] Goughari AS Mazhari S Pourrahimi AM Sadeghi MM Nakhaee N. Associations between components of metabolic syndrome and cognition in patients with schizophrenia. J Psychiatr Pract. (2015) 21:190–7. 10.1097/PRA.0000000000000065 25955261

[227] Takayanagi Y Cascella NG Sawa A Eaton WW. Diabetes is associated with lower global cognitive function in schizophrenia. Schizophr Res. (2012) 142:183–7. 10.1016/j.schres.2012.08.034 23031192 PMC4004180

[228] Broder JC Ryan J Shah RC Lockery JE Orchard SG Gilmartin-Thomas JF. Anticholinergic medication burden and cognitive function in participants of the ASPREE study. Pharmacotherapy. (2022) 42:134–44. 10.1002/phar.2652 34866212 PMC8863638

[229] Chatterjee S Bali V Carnahan RM Chen H Johnson ML Aparasu RR. Anticholinergic burden and risk of cognitive impairment in elderly nursing home residents with depression. Res Social Adm Pharm. (2020) 16:329–35. 10.1016/j.sapharm.2019.05.020 31182419

[230] Chatterjee S Talwar A Aparasu RR. Anticholinergic medications and risk of dementia in older adults: where are we now? Expert Opin Drug Saf. (2020) 19:1251–67. 10.1080/14740338.2020.1811227 32797761

[231] Campbell N Boustani M Limbil T Ott C Fox C Maidment I. The cognitive impact of anticholinergics: a clinical review. Clin Interv Aging. (2009) 4:225–33. 10.2147/CIA.S5358 19554093 PMC2697587

[232] Tsoutsoulas C Mulsant BH Kumar S Ghazala Z Voineskos AN Menon M. Anticholinergic burden and cognition in older patients with schizophrenia. J Clin Psychiatry. (2017) 78:e1284–e90. 10.4088/JCP.17m11523 29188908

[233] Eum S Hill SK Alliey-Rodriguez N Stevenson JM Rubin LH Lee AM. Genome-wide association study accounting for anticholinergic burden to examine cognitive dysfunction in psychotic disorders. Neuropsychopharmacology. (2021) 46:1802–10. 10.1038/s41386-021-01057-8 34145405 PMC8358015

[234] Joshi YB Thomas ML Hochberger WC Bismark AW Treichler EBH Molina J. Verbal learning deficits associated with increased anticholinergic burden are attenuated with targeted cognitive training in treatment refractory schizophrenia patients. Schizophr Res. (2019) 208:384–9. 10.1016/j.schres.2019.01.016 30738698 PMC8215853

[235] Vinogradov S Fisher M Warm H Holland C Kirshner MA Pollock BG. The cognitive cost of anticholinergic burden: decreased response to cognitive training in schizophrenia. Am J Psychiatry. (2009) 166:1055–62. 10.1176/appi.ajp.2009.09010017 19570929 PMC3735363

[236] Minzenberg MJ Poole JH Benton C Vinogradov S. Association of anticholinergic load with impairment of complex attention and memory in schizophrenia. Am J Psychiatry. (2004) 161:116–24. 10.1176/appi.ajp.161.1.116 14702259

[237] Schuster J-P Hoertel N von Gunten A Seigneurie A-S Limosin F. Benzodiazepine use among older adults with schizophrenia spectrum disorder: prevalence and associated factors in a multicenter study. Int Psychogeriatr. (2020) 32:441–51. 10.1017/S1041610219000358 31062670

[238] Bucci P Mucci A van Rossum IW Aiello C Arango C Baandrup L. Persistent negative symptoms in recent-onset psychosis: relationship to treatment response and psychosocial functioning. Eur Neuropsychopharmacol. (2020) 34:76–86. 10.1016/j.euroneuro.2020.03.010 32291210

[239] Marder SR Galderisi S. The current conceptualization of negative symptoms in schizophrenia. World Psychiatry. (2017) 16:14–24. 10.1002/wps.20385 28127915 PMC5269507

[240] Gaebel W Falkai P Hasan A. The revised German evidence- and consensus-based schizophrenia guideline. World Psychiatry. (2020) 19:117–9. 10.1002/wps.20706 31922675 PMC6953579

[241] Sanislow CA. RDoC at 10: changing the discourse for psychopathology. World Psychiatry. (2020) 19:311–2. 10.1002/wps.20800 32931117 PMC7491616

[242] Kotov R Jonas KG Carpenter WT Dretsch MN Eaton NR Forbes MK. Validity and utility of hierarchical taxonomy of psychopathology (HiTOP): I. Psychosis superspectrum. World Psychiatry. (2020) 19:151–72. 10.1002/wps.20730 32394571 PMC7214958

[243] First MB Gaebel W Maj M Stein DJ Kogan CS Saunders JB. An organization- and category-level comparison of diagnostic requirements for mental disorders in ICD-11 and DSM-5. World Psychiatry. (2021) 20:34–51. 10.1002/wps.20825 33432742 PMC7801846

[244] Lahey BB Moore TM Kaczkurkin AN Zald DH. Hierarchical models of psychopathology: empirical support, implications, and remaining issues. World Psychiatry. (2021) 20:57–63. 10.1002/wps.20824 33432749 PMC7801849

[245] Krueger RF Hobbs KA Conway CC Dick DM Dretsch MN Eaton NR. Validity and utility of hierarchical taxonomy of psychopathology (HiTOP): II. Externalizing superspectrum. World Psychiatry. (2021) 20:171–93. 10.1002/wps.20844 34002506 PMC8129870

[246] Giordano GM Bucci P Mucci A Pezzella P Galderisi S. Gender differences in clinical and psychosocial features among persons with schizophrenia: a mini review. Front Psychiatry. (2021) 12:789179. 10.3389/fpsyt.2021.789179 35002807 PMC8727372

[247] Giordano GM Stanziano M Papa M Mucci A Prinster A Soricelli A. Functional connectivity of the ventral tegmental area and avolition in subjects with schizophrenia: a resting state functional MRI study. Eur Neuropsychopharmacol. (2018) 28:589–602. 10.1016/j.euroneuro.2018.03.013 29653743

[248] Foussias G Siddiqui I Fervaha G Agid O Remington G. Dissecting negative symptoms in schizophrenia: opportunities for translation into new treatments. J Psychopharmacol. (2015) 29:116–26. 10.1177/0269881114562092 25516370

[249] Galderisi S Färden A Kaiser S. Dissecting negative symptoms of schizophrenia: history, assessment, pathophysiological mechanisms and treatment. Schizophr Res. (2017) 186:1–2. 10.1016/j.schres.2016.04.046 27185482

[250] Kirkpatrick B Fischer B. Subdomains within the negative symptoms of schizophrenia: commentary. Schizophr Bull. (2006) 32:246–9. 10.1093/schbul/sbj054 16492798 PMC2632226

[251] Konstantakopoulos G Ploumpidis D Oulis P Patrikelis P Soumani A Papadimitriou GN. Apathy, cognitive deficits and functional impairment in schizophrenia. Schizophr Res. (2011) 133:193–8. 10.1016/j.schres.2011.07.003 21788116

[252] Fusar-Poli P Papanastasiou E Stahl D Rocchetti M Carpenter W Shergill S. Treatments of negative symptoms in schizophrenia: meta-analysis of 168 randomized placebo-controlled trials. Schizophr Bull. (2015) 41:892–9. 10.1093/schbul/sbu170 25528757 PMC4466178

[253] Fleischhacker W Galderisi S Laszlovszky I Szatmári B Barabássy Á Acsai K. The efficacy of cariprazine in negative symptoms of schizophrenia: post hoc analyses of PANSS individual items and PANSS-derived factors. Eur Psychiatry. (2019) 58:1–9. 10.1016/j.eurpsy.2019.01.015 30738380

[254] Krause M Zhu Y Huhn M Schneider-Thoma J Bighelli I Nikolakopoulou A. Antipsychotic drugs for patients with schizophrenia and predominant or prominent negative symptoms: a systematic review and meta-analysis. Eur Arch Psychiatry Clin Neurosci. (2018) 268:625–39. 10.1007/s00406-018-0869-3 29368205

[255] Németh G Laszlovszky I Czobor P Szalai E Szatmári B Harsányi J. Cariprazine versus risperidone monotherapy for treatment of predominant negative symptoms in patients with schizophrenia: a randomised, double-blind, controlled trial. Lancet. (2017) 389:1103–13. 10.1016/S0140-6736(17)30060-0 28185672

[256] Mucci A Merlotti E Üçok A Aleman A Galderisi S. Primary and persistent negative symptoms: concepts, assessments and neurobiological bases. Schizophr Res. (2017) 186:19–28. 10.1016/j.schres.2016.05.014 27242069

[257] Kirkpatrick B Mucci A Galderisi S. Primary, enduring negative symptoms: an update on research. Schizophr Bull. (2017) 43:730–6. 10.1093/schbul/sbx064 28575513 PMC5472155

[258] Huhn M Nikolakopoulou A Schneider-Thoma J Krause M Samara M Peter N. Comparative efficacy and tolerability of 32 oral antipsychotics for the acute treatment of adults with multi-episode schizophrenia: a systematic review and network meta-analysis. Lancet. (2019) 394:939–51. 10.1016/S0140-6736(19)31135-3 31303314 PMC6891890

[259] Krause M Huhn M Schneider-Thoma J Bighelli I Gutsmiedl K Leucht S. Efficacy, acceptability and tolerability of antipsychotics in patients with schizophrenia and comorbid substance use. A systematic review and meta-analysis. Eur Neuropsychopharmacol. (2019) 29:32–45. 10.1016/j.euroneuro.2018.11.1105 30472164

[260] Galling B Roldán A Hagi K Rietschel L Walyzada F Zheng W. Antipsychotic augmentation vs. monotherapy in schizophrenia: systematic review, meta-analysis and meta-regression analysis. World Psychiatry. (2017) 16:77–89. 10.1002/wps.20387 28127934 PMC5269492

[261] Carpenter WT Jr. Heinrichs DW Alphs LD. Treatment of negative symptoms. Schizophr Bull. (1985) 11:440–52. 10.1093/schbul/11.3.440 2863871

[262] Siris SG Addington D Azorin JM Falloon IR Gerlach J Hirsch SR. Depression in schizophrenia: recognition and management in the USA. Schizophr Res. (2001) 47:185–97. 10.1016/S0920-9964(00)00135-3 11278136

[263] Kulhara P Avasthi A Chadda R Chandiramani K Mattoo SK Kota SK. Negative and depressive symptoms in schizophrenia. Br J Psychiatry. (1989) 154:207–11. 10.1192/bjp.154.2.207 2775947

[264] Siris SG. Depression in schizophrenia: perspective in the era of “atypical” antipsychotic agents. Am J Psychiatry. (2000) 157:1379–89. 10.1176/appi.ajp.157.9.1379 10964850

[265] Leucht S Arbter D Engel RR Kissling W Davis JM. How effective are second-generation antipsychotic drugs? A meta-analysis of placebo-controlled trials. Mol Psychiatry. (2009) 14:429–47. 10.1038/sj.mp.4002136 18180760

[266] Galling B Vernon JA Pagsberg AK Wadhwa A Grudnikoff E Seidman AJ. Efficacy and safety of antidepressant augmentation of continued antipsychotic treatment in patients with schizophrenia. Acta Psychiatr Scand. (2018) 137:187–205. 10.1111/acps.12854 29431197

[267] Helfer B Samara MT Huhn M Klupp E Leucht C Zhu Y. Efficacy and safety of antidepressants added to antipsychotics for schizophrenia: a systematic review and meta-analysis. Am J Psychiatry. (2016) 173:876–86. 10.1176/appi.ajp.2016.15081035 27282362

[268] Kirschner M Aleman A Kaiser S. Secondary negative symptoms - a review of mechanisms, assessment and treatment. Schizophr Res. (2017) 186:29–38. 10.1016/j.schres.2016.05.003 27230288

[269] Kirkpatrick B. Recognizing primary vs secondary negative symptoms and apathy vs expression domains. J Clin Psychiatry. (2014) 75:e09. 10.4088/JCP.13049tx3c 24813410

[270] Möller HJ. Clinical evaluation of negative symptoms in schizophrenia. Eur Psychiatry. (2007) 22:380–6. 10.1016/j.eurpsy.2007.03.010 17524626

[271] Carpenter WT. Primary psychosis: more to know, much more to do. World Psychiatry. (2021) 20:1–2. 10.1002/wps.20807 33432743 PMC7801827

[272] Galderisi S Giordano GM. We are not ready to abandon the current schizophrenia construct, but should be prepared to do so. Schizophr Res. (2022) 242:30–4. 10.1016/j.schres.2021.12.007 34924240

[273] Chekroud AM Bondar J Delgadillo J Doherty G Wasil A Fokkema M. The promise of machine learning in predicting treatment outcomes in psychiatry. World Psychiatry. (2021) 20:154–70. 10.1002/wps.20882 34002503 PMC8129866

[274] Giordano GM Pezzella P Perrottelli A Galderisi S. Die “Präzisionspsychiatrie” muss Teil der “personalisierten Psychiatrie” werden. Fortschr Neurol Psychiatr. (2020) 88:767–72. 10.1055/a-1211-2826 32869236

